# Smartphone-enabled video-observed versus directly observed treatment for tuberculosis: a multicentre, analyst-blinded, randomised, controlled superiority trial

**DOI:** 10.1016/S0140-6736(18)32993-3

**Published:** 2019-03-23

**Authors:** Alistair Story, Robert W Aldridge, Catherine M Smith, Elizabeth Garber, Joe Hall, Gloria Ferenando, Lucia Possas, Sara Hemming, Fatima Wurie, Serena Luchenski, Ibrahim Abubakar, Timothy D McHugh, Peter J White, John M Watson, Marc Lipman, Richard Garfein, Andrew C Hayward

**Affiliations:** a Institute of Health Informatics, University College London, London, UK; b Institute for Global Health, University College London, London, UK; c Centre for Clinical Microbiology, University College London, London, UK; d Research Department of Infection and Population Health, University College London, London, UK; e UCL Respiratory, Division of Medicine, University College London, London, UK; f Institute of Epidemiology and Health Care, University College London, London, UK; g Find and Treat, University College Hospitals NHS Foundation Trust, London, UK; h Royal Free London NHS Foundation Trust, London, UK; i MRC Centre for Outbreak Analysis and Modelling, Department of Infectious Disease Epidemiology School of Public Health, Imperial College London, London, UK; j NIHR Health Protection Research Unit in Modelling Methodology, Department of Infectious Disease Epidemiology School of Public Health, Imperial College London, London, UK; k Modelling and Economics Unit, National Infection Service, Public Health England, London, UK; l Division of Global Public Health, School of Medicine, University of California, San Diego, CA, USA

## Abstract

**Background:**

Directly observed treatment (DOT) has been the standard of care for tuberculosis since the early 1990s, but it is inconvenient for patients and service providers. Video-observed therapy (VOT) has been conditionally recommended by WHO as an alternative to DOT. We tested whether levels of treatment observation were improved with VOT.

**Methods:**

We did a multicentre, analyst-blinded, randomised controlled superiority trial in 22 clinics in England (UK). Eligible participants were patients aged at least 16 years with active pulmonary or non-pulmonary tuberculosis who were eligible for DOT according to local guidance. Exclusion criteria included patients who did not have access to charging a smartphone. We randomly assigned participants to either VOT (daily remote observation using a smartphone app) or DOT (observations done three to five times per week in the home, community, or clinic settings). Randomisation was done by the SealedEnvelope service using minimisation. DOT involved treatment observation by a health-care or lay worker, with any remaining daily doses self-administered. VOT was provided by a centralised service in London. Patients were trained to record and send videos of every dose ingested 7 days per week using a smartphone app. Trained treatment observers viewed these videos through a password-protected website. Patients were also encouraged to report adverse drug events on the videos. Smartphones and data plans were provided free of charge by study investigators. DOT or VOT observation records were completed by observers until treatment or study end. The primary outcome was completion of 80% or more scheduled treatment observations over the first 2 months following enrolment. Intention-to-treat (ITT) and restricted (including only patients completing at least 1 week of observation on allocated arm) analyses were done. Superiority was determined by a 15% difference in the proportion of patients with the primary outcome (60% *vs* 75%). This trial is registered with the International Standard Randomised Controlled Trials Number registry, number ISRCTN26184967.

**Findings:**

Between Sept 1, 2014, and Oct 1, 2016, we randomly assigned 226 patients; 112 to VOT and 114 to DOT. Overall, 131 (58%) patients had a history of homelessness, imprisonment, drug use, alcohol problems or mental health problems. In the ITT analysis, 78 (70%) of 112 patients on VOT achieved ≥80% scheduled observations successfully completed during the first 2 months compared with 35 (31%) of 114 on DOT (adjusted odds ratio [OR] 5·48, 95% CI 3·10–9·68; p<0·0001). In the restricted analysis, 78 (77%) of 101 patients on VOT achieved the primary outcome compared with 35 (63%) of 56 on DOT (adjusted OR 2·52; 95% CI 1·17–5·54; p=0·017). Stomach pain, nausea, and vomiting were the most common adverse events reported (in 16 [14%] of 112 on VOT and nine [8%] of 114 on DOT).

**Interpretation:**

VOT was a more effective approach to observation of tuberculosis treatment than DOT. VOT is likely to be preferable to DOT for many patients across a broad range of settings, providing a more acceptable, effective, and cheaper option for supervision of daily and multiple daily doses than DOT.

**Funding:**

National Institute for Health Research.

## Introduction

Directly observed treatment (DOT) has been the standard of care for tuberculosis since the early 1990s.^1^, ^2^ It arose from early observations that irregular treatment could threaten clinical outcomes and public health through generation of drug resistance, relapse, and transmission of infection.^3^ DOT is currently recommended by WHO^4^ and the American Thoracic Society.^5^ In England (UK) it is advised for patients at high risk of poor adherence,^6^ including those with clinically complex disease, multidrug-resistant (MDR) tuberculosis, mental health problems, previous tuberculosis treatment, or poor adherence.^6^ DOT is also advised for socially complex groups (eg, people with history of homelessness, imprisonment, or drug use or alcohol problems).

Although DOT can be administered in clinic, community, or home settings, it still entails substantial inconvenience to patients and service providers. 7-day treatment regimens are therefore generally administered through DOT 5 days per week and self-administered treatment at the weekend. Regimens given three times per week have also been approved with DOT and are used in England,^6^ although they are not currently recommended by WHO because of increased risk of treatment failure possibly because treatment is given less frequently.^4^

**Research in context**

**Evidence before this study**

We searched PubMed for studies published before 22 March, 2018, using the search terms “tuberculosis” AND (“video” OR “mobile”) AND “observe/observation”. 11 studies described implementation of synchronous (six) or asynchronous (five) video-observed therapy (VOT). Most studies were pilot projects assessing feasibility and acceptability of using VOT. They showed that VOT is an acceptable, flexible, cost-effective and patient-friendly intervention. Two cohort studies compared treatment observation levels for directly observed treatment (DOT) and synchronous VOT; one, a study in South Australia, reported 87·9% of treatment events observed for VOT and 68·9% for DOT; the second, a study in New York City, reported 95% adherence for VOT and 91% for DOT. No comparative studies have been published for asynchronous VOT.

**Added value of this study**

This is the first randomised controlled trial comparing the level of treatment observation for DOT and asynchronous VOT. A higher proportion of patients on VOT completed 80% or more scheduled treatment observations over the first 2 months following enrolment than those on DOT. The study also showed that VOT was cheaper to deliver than DOT.

**Implications of all the available evidence**

VOT is a more effective and cheaper approach to observation of tuberculosis treatment than DOT. The intervention has been acceptable when assessed in patients in various settings including North America, England (UK), Belarus, Kenya, and Vietnam.

Developments in video telephony technology have raised the possibility of remote video-observed treatment (VOT) as an alternative approach to DOT.^7^ Initially, this required a live video call (synchronous VOT) between the patient and observer.^8^, ^9^, ^10^, ^11^, ^12^, ^13^ More recently, smartphone apps have been developed that enable video clips to be recorded and forwarded for later viewing (asynchronous VOT).^14^ Asynchronous VOT is currently used in some clinics in the USA and has high reported levels of patient acceptability, decreased costs compared with DOT, and programmatic evidence of effectiveness.^14^, ^15^, ^16^, ^17^, ^18^ WHO therefore conditionally recommended VOT as an alternative to DOT in 2017, but the evidence was graded weak due to few randomised controlled trials available.^4^ Additionally, VOT has yet to be assessed in socially complex patients. Here, we report results from a randomised trial comparing treatment observation with asynchronous VOT versus in-person DOT for supporting treatment adherence in patients with active tuberculosis in England.

## Methods

### Study design and participants

We did a multicentre, analyst-blinded, randomised controlled, superiority trial at 22 clinics in England (UK; London [17 sites], Birmingham [three], Coventry [one], and Leicester [one]). Ethical approval was granted by the National Research Ethics Service Committee East of England—Essex, Research Ethics Committee, 20/03/2014, ref: 10/H0302/51. The full trial protocol is published on the International Standard Randomised Controlled Trial Number Registry).

Eligible patients were identified by case managers at each participating clinic and referred to the study team. Inclusion criteria included patients aged 16 years or older with active pulmonary or non-pulmonary tuberculosis who were eligible for DOT according to UK national guidance.^6^ Patients were invited to participate regardless of whether they had previously agreed to treatment observation. Patients were excluded if they were not suitable for VOT because they did not have access to facilities to charge a smartphone. They were also excluded if they had less than 2 months remaining on their treatment regimen, because the primary study outcome required measurement of adherence over 2 months. Patients with MDR tuberculosis were excluded because they require twice-daily treatment (these patients were recruited into a non-randomised study, that will be reported separately). All patients provided written informed consent to participate in the study.

### Randomisation and masking

We randomly assigned participants to either asynchronous VOT or DOT based in a clinic, community (eg, pharmacy or hostel), or home setting. Randomisation was provided by SealedEnvelope), a telephone and online software application used for randomly assigning patients in clinical trials. The system used randomisation by minimisation^19^ to ensure balance across study sites and the stage of treatment at the time of enrolment (ie, within the first 2 months of treatment and after the first 2 months of treatment). Minimisation allocates patients to best maintain balance in the stratification factors by calculating an imbalance score at each randomisation. It then assigns with higher probability each patient to the treatment that will reduce the imbalance.

### Procedures

DOT was delivered according to usual clinical practice.^6^ This involved treatment observation three to five times per week by a health-care or lay worker, with the remaining daily doses self-administered. VOT was provided by a centralised service in London (UK). Patients were trained to record and send videos of every dose ingested 7 days per week using a smartphone app developed by researchers at the University of California (San Diego, CA, USA).^14^ Trained treatment observers viewed the videos through a password-protected website. As a safety precaution, to address concerns that reduced face-to-face contact might lead to side-effects being undetected, patients were also encouraged to report adverse drug events on the videos. Smartphones (Samsung Galaxy S3/S4/Xcover3) and data plans (including UK calls and texts) were provided free of charge, paid by the study at commercial rates. Patients signed a form agreeing to return the phone at the end of treatment (details on returning the phones in the appendix). DOT and VOT observation records were completed by observers until treatment or study end. Full details of interventions are in the appendix.

### Outcomes

The primary outcome was successful completion of 80% or more of scheduled treatment observations in the 2 months following randomisation. The proportion of scheduled observations (measured on a continuous scale) successfully completed in the 2 months following enrolment, and throughout treatment, was a secondary outcome.

Other secondary outcome measures were: sputum culture results at 2 months post-treatment initiation; treatment outcomes; occurrence of adverse events; numbers of hospitalisations; staff time spent observing or travelling to observe patients; staff time and cost of travel when re-engaging patients; cost of treatment observation; patient satisfaction, resource use, and health-related quality of life.

Semi-structured interviews were done with 16 patients selected to represent a range of backgrounds and VOT and DOT successes and failures. Full results of the qualitative analysis will be reported separately. Details of the methods used for collection of the additional outcomes are in the appendix.

### Statistical analysis

We determined that a sample of 200 patients per arm would provide a power of 90% to detect a 15% difference in the proportion of patients with the primary outcome (60% *vs* 75%). This was based on a two-sided significance level of 5%. Following a review of study progress, the funder's study monitoring committee requested an interim analysis, the plan for which was published on the International Standard Randomised Controlled Trial Number Registry before analysis. It included a stopping rule using the Haybittle–Peto boundary of 0·001 for the primary outcome. We used the approach described in the interim analysis plan for the final analysis. This involved main and sensitivity analyses for intention-to-treat (ITT) and restricted groups, described below and summarised in the appendix.

In the main analysis, VOT treatment observations were classified as successfully completed if ingestion of all medicines was observed, or if video clips were received but not viewable because of a technical complication (since patients had no control over whether videos were corrupted). The sensitivity analysis considered only videos for which all medicines were observed as successfully completed. The ITT analysis included all patients, analysed according to the arm to which they were originally randomised. The restricted analysis excluded patients with less than 1 week of observation in the allocated arm. This was designed to include only those patients who had, at least initially, engaged with the allocated intervention.

We used logistic regression to analyse the primary outcome and linear regression for the secondary outcomes. Time since start of treatment, age, and sex were considered a priori as potential confounders and included in all models. For the restricted analysis, we also considered covariates that might have affected initial engagement with the allocated intervention (homelessness, imprisonment, drug use, alcohol problems, immigration concerns, mental health problems, previous loss to follow-up, and no recourse to public funds). All analyses accounted for clustering at the level of the clinic using robust standard errors.^20^ Likelihood ratio tests for interaction were used to assess evidence of differing effect size in population subgroups. Analyses were done using Stata (version 14) and R (version 3.3.2) software. This trial is registered with the International Standard Randomised Controlled Trials Number registry, number ISRCTN26184967.

### Role of the funding source

The funder of the study had no role in study design, data collection, data interpretation, or writing of the report. The corresponding author had full access to all the data in the study and had final responsibility for the decision to submit for publication.

## Results

**Figure 1:**
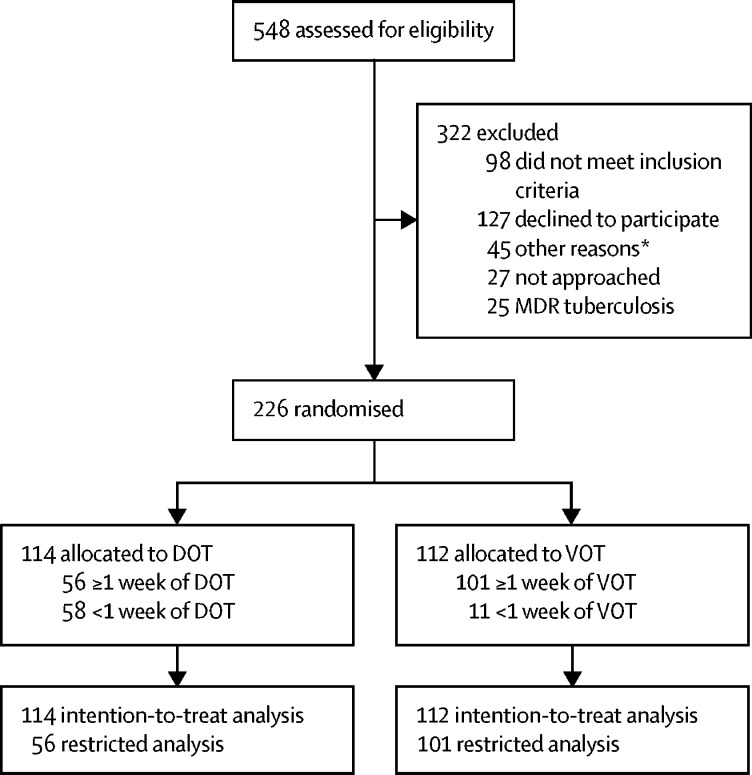
Enrolment and randomisation MDR=multi-drug resistant. *The most common other reason for not enrolling patients (32 of 45) was clinic staff considering that the patient needed intensive face-to-face support for emotional, medical, or physical reasons, or because of imminent risk of loss to follow-up. Characteristics of eligible patients who were approached but refused to take part in the study are in the appendix.

Between Sept 1, 2014, and Oct 1, 2016, we screened 548 patients with tuberculosis for eligibility. Of these, 322 (59%) were ineligible and 226 (41%) were randomly assigned to the study groups (figure 1). Follow-up continued until Dec 31, 2016. ITT analyses included 112 to VOT and 114 patients assigned to DOT.

**Figure 2:**
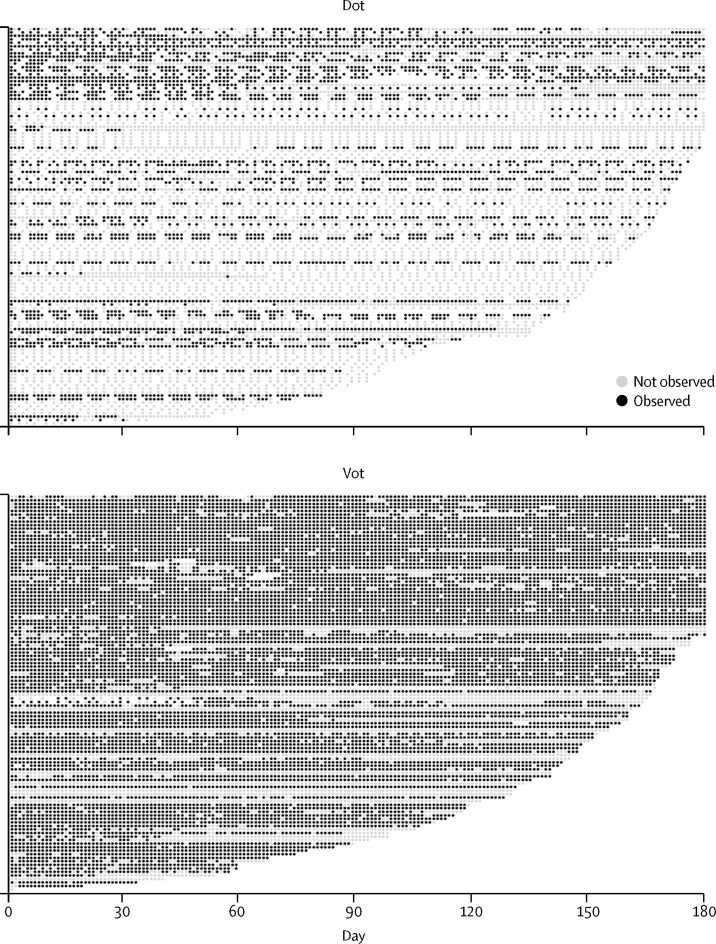
Level of observation Each row represents one patient. Each dot represents one scheduled treatment observation day. Observed (black) and unobserved (grey) scheduled doses are shown for each patient in the study through the course of follow-up. Patients are ordered according to their length of treatment time remaining after randomisation.

Patients were mainly young adults born outside the UK (table 1). A high proportion had a history of homelessness, imprisonment, drug use, alcohol problems, or mental health problems. Baseline characteristics were similar between the two groups. Patients were substantially more likely to engage initially (ie, have at least 1 week of follow-up) with VOT (101 [90%] of 112) than DOT (56 [49%] of 114). Levels of initial engagement with VOT exceeded 70% in all subgroups, but with DOT were particularly low (<50%) in younger adults, foreign-born patients and those without social risk factors or mental health problems (table 1). Amongst the 56 patients who initially engaged with DOT, 27 (48%) had home-based DOT, 20 (36%) clinic-based, and nine (16%) community based (eg, a local pharmacy). DOT was scheduled three times per week for 14 patients, and five times per week for the remainder. The level of observation achieved in each study arm is in figure 2. In the ITT analysis, 78 (70%) of 122 patients on VOT successfully achieved the primary outcome (≥80% scheduled observations successfully completed during the first 2 months), compared with 35 (31%) of 114 patients on DOT: partially adjusted odds ratio (OR) 5·48, 95% CI, 3·10–9·68; p<0·0001 (table 2).

**Table 1:** Baseline characteristics of the study population

	**Directly observed treatment**		**Video-observed therapy**	
	As randomised (n=114)	Restricted* (n=56)	As randomised (n=112)	Restricted* (n=101)
**Age (years)**				
16–34	61 (54%)	27 (48%)	64 (57%)	58 (57%)
35–54	45 (40%)	22 (39%)	35 (31%)	32 (32%)
≥55	8 (7%)	7 (13%)	13 (12%)	11 (11%)
**Sex**				
Male	83 (73%)	42 (75%)	82 (73%)	73 (72%)
Female	31 (27%)	14 (25%)	30 (27%)	28 (28%)
**Born in the UK**				
No	83 (73%)	37 (66%)	93 (83%)	85 (84%)
Yes	31 (27%)	19 (34%)	19 (17%)	16 (16%)
**Previous tuberculosis**				
No	82 (72%)	40 (71%)	85 (76%)	75 (74%)
Yes	30 (26%)	15 (27%)	27 (24%)	26 (26%)
**Pulmonary**				
No	41 (36%)	19 (34%)	43 (38%)	39 (39%)
Yes	73 (64%)	37 (66%)	69 (62%)	62 (61%)
**Social risk factor**†				
Never	48 (42%)	15 (27%)	47 (42%)	44 (44%)
>5 years ago	19 (17%)	10 (18%)	19 (17%)	16 (16%)
Within 5 years	47 (41%)	31 (55%)	46 (41%)	41 (41%)
**Homeless**				
Never	77 (68%)	31 (55%)	70 (63%)	64 (63%)
>5 years ago	14 (12%)	10 (18%)	16 (14%)	15 (15%)
Within 5 years	23 (20%)	15 (27%)	24 (21%)	20 (20%)
**Prison**				
Never	93 (82%)	44 (79%)	97 (87%)	89 (88%)
>5 years ago	9 (8%)	7 (13%)	8 (7%)	6 (6%)
Within 5 years	11 (10%)	4 (7%)	7 (6%)	6 (6%)
**Drug use**				
Never	96 (84%)	44 (79%)	89 (80%)	82 (81%)
>5 years ago	2 (2%)	2 (4%)	4 (4%)	3 (3%)
Within 5 years	15 (13%)	10 (18%)	18 (16%)	15 (15%)
**Alcohol problems**				
No	91 (80%)	38 (68%)	92 (82%)	83 (82%)
Yes	21 (18%)	18 (32%)	17 (15%)	15 (15%)
**Mental health problems**				
No	94 (83%)	44 (79%)	94 (84%)	87 (86%)
Yes	18 (16%)	12 (21%)	14 (13%)	10 (10%)

Data shown by allocated intervention and initial engagement (at least 1 week on allocated intervention).

* Initial engagement with intervention (at least 1 week of observation in allocated arm).

† History of homelessness, imprisonment, drug use or alcohol problems, or mental health problems.

**Table 2:** Numbers and proportions of patients and results of logistic regression analysis for the primary outcome

	n (%) with primary outcome*		**Unadjusted**		**Partially adjusted**†		**Fully adjusted**	
	DOT	VOT	OR (95% CI)	p value	Adjusted OR (95% CI)	p value	Adjusted OR† (95% CI)	p value
**Intention to treat**								
Total	114	112	..	..	..	..	..	..
Main‡	35 (31%)	78 (70%)	5·18 (2·94–9·12)	<0·0001	5·48 (3·10–9·68)	<0·0001	..	..
Sensitivity	35 (31%)	68 (61%)	3·49 (2·01–6·04)	<0·0001	3·60 (1·91–6·79)	<0·0001	..	..
**Restricted**§								
Total	56	101	..	..	..	..	..	..
Main	35 (63%)	78 (77%)	2·03 (1·00–4·15)	0·051	2·23 (1·16–4·27)	0·016	2·52 (1·17–5·47)	0·019
Sensitivity	35 (63%)	68 (67%)	1·24 (0·62–2·45)	0·542	1·29 (0·71–2·34)	0·398	1·44 (0·75–2·75)	0·273

Data shown by trial arm for intention-to-treat and restricted analyses. DOT=directly observed treatment. VOT=video-observed therapy. OR=odds ratio.

* Primary outcome: patients who had ≥80% observations successfully completed in the first 2 months following randomisation.

† Partially-adjusted models adjusted for time since start of treatment, age, sex, and treatment. Fully-adjusted models (for restricted analysis only) additionally adjusted for current social risk factor (homelessness, imprisonment, drug use, alcohol problems, immigration concern), ever lost to follow-up, no recourse to public funds, mental health problems.

‡ Main analysis: VOT treatment observations were classified as successfully completed if ingestion of all medicines was observed, or if video clips were received but not viewable due to a technical complication. Sensitivity analysis: only videos for which ingestion of all medicines was observed were classified as successfully completed.

§ Restricted analysis included only patients who engaged initially (had at least 1 week of observation) on the allocated treatment arm.

For the secondary outcome of the proportion of scheduled observations successfully completed over the first 2 months, in the ITT analysis, 5091 (79%) of 6474 scheduled observations were successfully completed on VOT, compared with 1774 (45%) of 3922 on DOT. The mean proportions of doses observed per patient were 78% (41%) for VOT and 36% (SD 31%) for DOT (adjusted mean difference in proportions 41%, 95% CI 29–53; p<0·0001). In the restricted analysis, the overall proportions were 5091 (86% [SD 17%]) of 5893 for VOT and 1774 (73% [27%]) of 2418 for DOT (adjusted mean difference in proportions 14%, 95% CI 7–20; p<0·0001). Full results for this secondary outcome are in the appendix.

**Figure 3:**
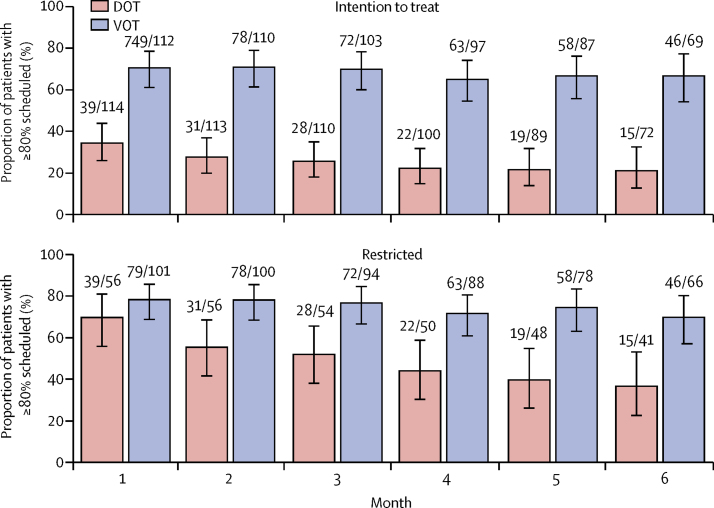
Proportions of patients with 80% or more of scheduled doses observed through treatment Numbers above bars are the numbers of patients who had scheduled treatment observations in each month following randomisation and the numbers who completed 80% or more scheduled observations. Error bars are 95% CIs.

High observation rates were maintained in the VOT arm, but they rapidly decreased in the DOT arm (figure 3). Over the full follow-up period (up to 6 months) 12 422 (77%; 95% CI 76–77) of 16 230 scheduled observations were completed in the VOT arm compared with 3884 (39%; 38–40) of 9882 scheduled observations in the DOT arm (p<0·0001). In the restricted analysis, over the full follow-up period 12 422 (83%; 83–84) of 14 907 of scheduled observations were completed in the VOT arm compared with 3884 (61%; 60–62) of 6351 in the DOT arm (p<0·0001). Observation completion rates were higher for VOT than DOT in all subgroups (table 3). There were no significant differences in positive sputum cultures at 2 months following treatment onset, treatment completion, loss to follow-up or numbers of hospital admissions between trial arms (appendix). 368 adverse events were reported by 32 patients on VOT and 184 reported by 15 on DOT (table 4). 169 unscheduled outpatient appointments were made by 47 patients on VOT, compared with 233 by 42 patients on DOT.

**Table 3:** Numbers and proportions of patients with more than 80% of scheduled doses observed over the first 2 months

		**DOT**		**VOT**		**p_interaction_**
		n	≥80% observed (%)	n	≥80% observed (%)	
Total		114	35 (31%)	112	78 (69%)	..
Age group (years)						
	16–34	61	15 (25%)	62	45 (73%)	0·439
	35–54	45	16 (36%)	35	25 (71%)	..
	≥55	8	4 (50%)	13	8 (62%)	..
Sex						
	Male	83	29 (35%)	82	60 (73%)	0·363
	Female	31	6 (19%)	30	18 (60%)	..
Born in the UK						
	No	83	22 (27%)	93	66 (71%)	0·430
	Yes	31	13 (42%)	19	12 (63%)	..
Previous tuberculosis						
	No	82	25 (31%)	85	58 (68%)	0·463
	Yes	30	9 (30%)	27	20 (74%)	..
	Unknown	2	1 (50%)	0	..	..
Site of disease						
	Pulmonary only	60	20 (33%)	55	37 (67%)	0·662
	Pulmonary and extrapulmonary	13	5 (39%)	14	11 (79%)	..
	Extrapulmonary only	41	10 (24%)	43	30 (70%)	..
Social risk factor (any)*						
	Never	48	8 (17%)	47	35 (75%)	0·0808
	>5 years ago	19	6 (32%)	19	11 (58%)	..
	Within 5 years	47	21 (45%)	46	32 (70%)	..
Homeless						
	Never	77	19 (25%)	70	49 (70%)	0·179
	>5 years ago	14	7 (50%)	16	10 (63%)	..
	Within 5 years	23	9 (39%)	24	17 (71%)	..
	Unknown	0	..	2	2 (100%)	..
Prison						
	Never	93	26 (28%)	97	68 (70%)	0·443
	>5 years ago	9	5 (56%)	8	5 (63%)	..
	Within 5 years	11	3 (27%)	7	5 (71%)	..
	Unknown	1	1 (100%)	0	..	..
Drug use						
	Never	96	27 (28%)	89	65 (73%)	0·606
	>5 years ago	2	2 (100%)	4	3 (75%)	..
	Within 5 years	15	6 (40%)	18	9 (50%)	..
	Unknown	1	0	1	1 (100%)	..
Alcohol problems						
	No	91	22 (24%)	92	66 (72%)	0·545
	Yes	21	13 (62%)	17	12 (71%)	..
	Unknown	2	0	3	0	..
Mental health problems						
	No	94	28 (30%)	94	67 (71%)	0·866
	Yes	18	7 (39%)	14	8 (57%)	..
	Unknown	2	0	4	3 (75%)	..
Immigration concerns						
	No	102	32 (31%)	99	70 (71%)	0·761
	Yes	9	2 (22%)	9	5 (56%)	..
	Unknown	3	1 (33%)	4	3 (75%)	..

Data shown by trial arm and baseline characteristics. DOT=directly observed treatment. VOT=video-observed therapy.

* History of homelessness, imprisonment, drug use or alcohol problems, or mental health problems

**Table 4:** Adverse events by trial arm

	**Number of reports**		**Number of patients**	
	DOT	VOT	DOT (n=114)	VOT (n=112)
Stomach pain, nausea, or vomiting	82	73	9 (8%)	16 (14%)
Eye problems	0	7	0	4 (4%)
Pain or swelling in face or joints	0	27	0	5 (4%)
Numbness, pain, or tingling in hands or feet	0	21	0	4 (4%)
Skin rash, severe itching, or hives	39	55	2 (2%)	6 (5%)
Headache or dizziness	9	21	2 (2%)	7 (6%)
Fever or chills	0	2	0	1 (<1%)
Unusual tiredness or loss of appetite	25	18	5 (4%)	4 (4%)
Other pain	0	144	0	13 (12%)
Not specified	29	0	3 (3%)	0

DOT=directly observed treatment. VOT=video-observed therapy.

Average staff time per dose observed was 56 min (SD 54) for community based DOT (including travel time); 15 min (12) for clinic-based DOT, and 3·2 min (0·5) for VOT. Patients on DOT spent a mean 29 min (SD 48) per week on treatment observation (including travelling to or from clinics, waiting for appointments, and appointment time). Those on VOT spent a mean of 1·8 min (2·2) setting up and recording each video.

The costs of providing DOT over 6 months were estimated at £5700 per patient for observations five times per week, and £3420 for observations three times per week. For daily VOT over 6 months, costs were estimated at £1645 per patient (in a service managing 50 patients; appendix).

The sensitivity analysis excluding corrupted videos showed similar effects (table 2), as did the main restricted analysis.

## Discussion

In this trial, VOT enabled higher levels of treatment observation for patients with tuberculosis, both over the first 2 months of treatment and throughout treatment, than DOT. VOT also supported daily dosing, was effective for socially complex populations, and had a lower drop-out rate than DOT. The absence of face-to-face contact did not reduce the identification of adverse events or lead to more unscheduled appointments. VOT reduced staff time requirements, especially compared with home-based DOT, making VOT cheaper than DOT even after taking into account the costs of the telephones and data plans provided by the study.

Systematic reviews and meta-analyses draw differing conclusions about the effectiveness of DOT.^21^, ^22^ However, one review showed that DOT increased treatment success, adherence, and 2-month sputum conversion, and decreased loss to follow-up and acquired drug resistance compared with self-administered treatment.^4^ Community based DOT was more effective than hospital-based or clinic-based DOT, highlighting the importance of making DOT convenient for patients. The review also showed that DOT was more effective when delivered by health staff or lay workers than by family members.^4^ VOT provides the technology to support professional treatment observation that is more convenient and cheaper than in-person DOT. In this study, VOT had a much higher uptake rate than DOT (91% *vs* 46%). This is a clear indication that providing treatment observation in a more convenient, flexible, and less intrusive way makes VOT a more acceptable mode of treatment for many patients.

VOT, as used in this study, has a wide range of components beyond convenience of observation. The intervention included personal support, where patients met the VOT observer for training, and received regular personalised messages as reminders, confirmation of receipt of video clips, or to follow up when clips were not received. Patients were specifically asked to report any adverse events after each clip and the observers also supported onward referral to deal with reported adverse events. Numerically more adverse events were reported in the VOT group than in the DOT group, which was likely due to the systematic reporting, although it could also have resulted from better compliance. Patients were provided with a smartphone with a data plan, and free domestic calls and text messages. This acted facilitated easy communication and improved access to care providers, and was a material incentive that was valued by patients although patients were asked to return the smartphone on completion of the study. On average, fewer than one phone was needed per patient because phones were re-used throughout the trial.

The trial had several limitations. It was not possible to blind patients or treatment observers to the intervention, although the investigators and statistician were blinded during the analysis. We could not distinguish between doses that were not observed and doses that were not taken. Case managers reported that many of the unobserved scheduled doses for DOT had previously been negotiated with clinic case managers because of patients having conflicting appointments or priorities, or case managers choosing to trust patients to self-administer scheduled doses. Approximately 7% of video clips submitted during the first 2 months of treatment were corrupted because of a software bug that was subsequently fixed (appendix). In our main analysis we assumed that submission of a corrupted clip represented pill ingestion since patients were not aware that the videos they submitted were corrupted. We tested the potential impact of this assumption in sensitivity analysis A (appendix) which regarded corrupted clips as unobserved. This sensitivity analysis produced similar results to the main analysis.

ORs reported through logistic regression should not be regarded as accurate estimates of relative risk. For example, our adjusted OR for the main analysis is 5·48 but the corresponding unadjusted relative risk would be 2·27. The primary outcome (observing 80% scheduled doses) could be considered to be biased in favour of DOT, because it required substantially more VOT doses (scheduled 7 days per week) to be observed than DOT doses (scheduled three or five times per week). The restricted analysis further favoured DOT, because it included only the highly selected subset of patients allocated to DOT who were willing to be observed. That VOT still outperformed DOT under these constraints, especially when considered over 6 months, adds to the robustness of the findings.

The study was not powered to detect differences in culture conversion rate, treatment completion, loss to follow-up, relapse, or development of drug resistance. Nevertheless, it is reasonable to assume that improved adherence might improve all these outcomes. VOT might also improve treatment outcomes and reduce the risk of adverse events compared with DOT, because clinicians do not need to schedule intermittent high dose regimens to ensure observation.

Another limitation was the exclusion of patients with MDR tuberculosis from the randomised trial. This was done because these patients had a range of treatment regimens, some of which included injectable medications or multiple scheduled doses to be observed per day. It would be impractical for all these observations to be made through face-to-face DOT and we therefore did not consider there to be equipoise between VOT and DOT for these individuals. Patients with MDR tuberculosis were recruited into a separate non-randomised arm of the study that will be reported separately.

The patients in this study were distributed across multiple sites and had many complex social needs. The findings are therefore likely to be generalisable to similar settings where patients with risk factors for non-adherence are offered support. Although the study was done in a low-incidence, high-income setting, we think it plausible that the intervention would also be effective in high-incidence, low-income settings, provided that good internet and cellular data connections are available, as is the case in many urban areas in LMICs. Relative and absolute costs of smartphones, data plans, and staff time differ between countries, but the large savings in staff time mean that VOT is likely to be cheaper than DOT in such settings. There is a need for more research in this area, including comparative studies between different digital adherence interventions in high-burden settings to measure effectiveness, feasibility with respect to technological infrastructure, and acceptability and cultural appropriateness of the interventions. Given the effectiveness of VOT for patients with complex social needs, the intervention could also be effective in treating other conditions that are prevalent in these populations; eg, hepatitis C.

There is an urgent global need for more effective and cheaper alternatives to DOT to enable effective ambulatory care of both drug-sensitive and MDR tuberculosis. In particular, it is important that new opportunities for shorter regimens for MDR tuberculosis are not lost because of insufficient attention to adherence.^23^, ^24^ WHO now recommends that VOT can be a suitable alternative to DOT and has published guidance on its implementation.^4^ VOT is, in fact, likely to be preferable to DOT for many patients across a broad range of settings, providing a more acceptable, effective, and cheaper option for supervision of daily and multiple daily doses than DOT.

Based on the experience of this trial, VOT has been adopted by the UK National Health Service in London (UK), provided by the Find and Treat service. Most patients with MDR tuberculosis in London are now treated using VOT, and many of these patients require multiple daily dosing (Joe Hall, Find and Treat, personal communication). The intervention has also been used successfully in children aged as young as 12 years and is being provided to an increasing number of patients outside London, particularly those with complex social needs. In settings such as this where mobile-internet connectivity makes VOT feasible, it is likely to make an important contribution to tuberculosis eradication.
