# Supplementary material for: Smartphone-enabled video-observed versus directly observed treatment for tuberculosis: a multicentre, analyst-blinded, randomised, controlled superiority trial
Source: Lancet. 2019 Mar 23;393(10177):1216–24. doi: 10.1016/S0140-6736(18)32993-3 (PMC6429626; doi:10.1016/S0140-6736(18)32993-3)
Supplement: Supplementary appendix [file mmc1.pdf]

THE LANCET

Supplementary appendix

This appendix formed part of the original submission and has been peer reviewed. We post it as supplied by the authors.

Supplement to: Story A, Aldridge RW, Smith CM, et al. Smartphone-enabled video-observed versus directly observed treatment for tuberculosis: a multicentre, analyst-blinded, randomised, controlled superiority trial. *Lancet* 2019; published online Feb 21. http://dx.doi.org/10.1016/S0140-6736(18)32993-3.

APPENDIX

Smartphone-enabled video observed versus directly observed treatment for tuberculosis: a randomised controlled trial

CONTENTS

ADDITIONAL METHODS 3
Interventions 3
Directly observed treatment (DOT) 3
Video observed treatment (VOT) 3
Intervention cross-over 3
Additional care 3
Patient information 4
Follow-up 4
Stopping rules 4
Classification of observation time 4
Sensitivity analyses 4
Collection of additional outcomes 4
Costs 5
ADDITIONAL RESULTS 6
Sensitivity analyses 6
Sputum smear tests 6
Treatment completion 6
Hospitalisations and outpatient appointments 6
Patient satisfaction 6
Health-related quality of life 6
Participant resource use 6
Observer resource use 6
Costs 7
TABLE S1: Analyses conducted 8
TABLE S2: Costs of VOT. 9
TABLE S3: Characteristics of eligible patients approached to take part in the trial who were recruited or who refused. 10
TABLE S4: Observation at two months (total doses observed). 11
TABLE S5: Observation at two months (primary outcome). 12
TABLE S6: Observation at two months (secondary outcome). 13
TABLE S7: Trial outcome of DOT by DOT location. 14
TABLE S8: Treatment outcome by trial arm. 15
TABLE S9: Patient satisfaction by trial arm. 16
TABLE S10: Health-related quality of life for patients on DOT and VOT, measured by time trade-off (TTO) derived from EQ5D-3L using UK value set 17
TABLE S11: Health-related quality of life for patients on DOT and VOT, measured by EQ5D-3L visual analogue scale 18
TABLE S12: DOT participant resource use, by survey time* 19
TABLE S13: VOT participant resource use, by survey time* 20
TABLE S14: Lost income and missed time from paid/ unpaid work, by survey time and trial arm* ... 21
TABLE S15: Clinic-based DOT observer resource use, by survey time 22

TABLE S16: Community-based DOT observer resource use, by survey time 23
TABLE S17: VOT observer resource use, by survey time 24
TABLE S18: Costs per patient of DOT and VOT, by treatment duration. 25
FIGURE S1: Proportion of clips with technical issues during the study. 26
FIGURE S2: Distribution of number of clips with technical issues by patient. 26
SPIRIT PROTOCOL 27
Administrative information 29
Introduction 31
Methods: Participants, interventions, and outcomes 32
Methods: Data collection, management, and analysis 43
Monitoring 48
Ethics and dissemination 49
Appendices 51
STATISTICAL ANALYSIS PLAN 53
ADDITIONAL REFERENCES 60

ADDITIONAL METHODS

Interventions

Directly observed treatment (DOT)

DOT was delivered according to usual clinical practice (three to five times weekly, delivered by a healthcare/ lay worker in clinic, community or home settings). Clinics were permitted to provide incentives and enablers, such as travel costs, as per normal practice. Clinic case managers would attempt to re-engage patients who failed to attend DOT sessions.

Video observed treatment (VOT)

Patients were issued with an Android smartphone with a monthly 5 gigabyte data plan including unlimited domestic calls and text messages. The smartphone had a pre-loaded VOT app developed at the University of California San Diego. The VOT app enabled patients to film themselves taking their treatment with the resultant video clips being automatically encrypted and uploaded to a secure National Health Service (NHS) approved server when the phone was connected to a cellular or Wi-Fi network.

VOT observers (trained members of the study team) accessed video clips via a password protected patient administration website. All VOT patients were on daily treatment schedules and were asked to video record every treatment dose using the app. Weekday doses were read on the same day, with evening doses read the next day, except for Friday evenings, which were read the following Monday. Weekend doses were read on Monday morning.

The person who would be observing their videos trained the patients, where possible. Training was done with the aid of sample video that could be played on the smartphone. During training patients were asked to take each pill separately so ingestion could be clearly seen and, at the end of dosing, to drink water from a clear glass and then to show their mouth was empty by opening their mouth and protruding their tongue.

Patients could opt to receive daily SMS reminders, and videos were regularly acknowledged with a personalised, motivational text message or email written after reading the clip. If video clips were not submitted the observer would attempt to contact the patient by telephone. If they could not be contacted within 24 hours then the patient’s clinic case manager was contacted.

Patients were asked to report any adverse events on the video clip. A study nurse assessed any reports of adverse events and referred patients to clinic if they considered these needed follow-up.

Patients were allowed to use the phone for UK calls, text messages, emails and internet access but no other incentives were provided. Patients signed a form agreeing to return the phone at the end of treatment. Approximately 40% participants did not return phones on request (reasons given included that they had lost the phones; felt that they had “earned” them; or that it would be inconvenient because they had their contacts on them). At the end of the study, returned phones were donated to the Find & Treat service, which is running the VOT programme.

Intervention cross-over

If patients had two episodes of non-adherence they were switched into the other trial arm. Four patients crossed-over from VOT to DOT; and five from DOT to VOT. For the purposes of this analysis, all doses following cross-over were considered unobserved.

Additional care

Patients’ clinical care remained the responsibility of the collaborating clinic. This involved regular outpatient appointments (initially monthly, then with decreasing frequency according to usual clinical practice). Clinics could also refer patients in either arm to relevant agencies to address social/ addiction issues. The use of dosset boxes as a patient medication organiser was allowed.

Patient information

The information provided prior to consent included highlighting that their clinic recommended them to have their treatment observed and that if they consented to the study they would be randomised to an offer of VOT or DOT but they were free to refuse observation at any point.

Follow-up

A DOT diary was maintained by clinics or community DOT observers with the observer signing for each scheduled dose. This was faxed to the study team on a weekly basis. VOT observers recorded treatment observations on the VOT patient administration system. Follow up continued until the earliest of end of treatment date or study end date (December 31st 2016). If patients were lost to follow-up care, their planned subsequent doses were assumed to have been unobserved.

Stopping rules

As this was a non-pharmaceutical trial we did not develop *a priori* stopping rules. However, in view of a decline in tuberculosis incidence leading to slower than expected recruitment and clear indications that many patients refused DOT or had less than one week of direct observation following randomisation, the funders of the trial requested an interim analysis to inform whether or not the trial should continue recruitment. An analysis plan was developed prior to this interim analysis including a stopping rule using the Haybittle–Peto boundary of 0.001 for the primary outcome. This analysis plan was published prior to analysis on the International Standard Randomised Controlled Trial Number Registry (study ISRCTN26184967, DOI 10.1186/ISRCTN26184967).

Classification of observation time

Doses occurring during periods when patients were in hospital or prison were considered as successfully observed in both arms. Time spent outside the UK was excluded from observation time. Any doses subsequent to cross-over of treatment arms were considered unobserved.

Sensitivity analyses

In the main analysis, VOT treatment observations were classified as successfully completed if all medicines had been observed, or if video clips had been received but were not viewable because they were corrupted (as patients had no control over whether videos were corrupted).

We also conducted two sensitivity analyses. Sensitivity Analysis A considered only videos for which all medicines were observed as successfully completed. In addition to the clips considered as successfully completed in the main analysis, Sensitivity Analysis B considered any other technical issues with VOT clips as successfully completed; and self-administered treatment as successfully completed for DOT.

Collection of additional outcomes

For patients recruited prior to the end of the two month initiation phase, clinics were asked to take sputum samples from patients at the first appointment after the end of the initiation phase for culture testing. VOT and DOT observers completed surveys at two months and six months to record the amount of time spent observing medication, travelling to observe medication, time spent reengaging patients with the intervention, travelling to reengage patients, money spent on travel, and the number of outpatient appointments and hospitalisations.

We conducted telephone interviews with patients to measure levels of patient satisfaction with their mode of observation at two and six months. In these surveys, patients were also asked to complete health related quality of life (EQ5D-3L) questionnaires, and to report the time and money spent travelling to appointments, time waiting in clinic and in the appointment (DOT); or time spent setting up, filming and submitting video clips (VOT). They were also asked to report any lost income or missed time from paid/unpaid work as a result of treatment observation.

Semi-structured qualitative interviews were conducted with a selection of 16 patients selected to represent a range of backgrounds, VOT and DOT successes and failures.

Treatment outcome data were derived from the London Tuberculosis Register and contact with case managers at the end of the study.

Costs

Taking the perspective of the NHS, DOT involving observations 3 times weekly costs £570 per patient per month, based on costs calculated by White et al. 2011(1) and inflated to 2015-2016 prices using the hospital & community health services (HCHS) index.(2) Observation 5 times weekly costs £950 per patient per month. All of the costs of DOT are unit costs, and therefore the cost of DOT per patient is determined only by the duration of treatment.

Sources of data used to calculate costs for VOT are shown in Table S2. For VOT, there is an initial IT infrastructure set-up cost of £2,000, and a monthly cost of cloud data storage, software licences, and system maintenance of £3,270. Providing VOT requires a band 7 nurse to lead the service, conduct face-to-face training with patients, and liaise with tuberculosis clinics. The monthly cost of a 100% FTE band 7 nurse in Inner London is £4,425.

Observation of videos and patient support is provided by band 5 nurses. A patient creates 7 videos per week. With 20% of videos being checked for quality assurance, each patient requires 8.4 observations per week. A nurse can observe 65 videos per day (based on this study), which is 325 videos per week and therefore each full-time equivalent (FTE) nurse can manage 38 patients at once, i.e. each patient requires 2·6%FTE of a nurse. The monthly cost of a 100%FTE band 5 nurse in Inner London is £3,045, of which 2·6% is £79.

ADDITIONAL RESULTS

Sensitivity analyses

Sensitivity analyses showed similar results to the main analysis for the ITT analysis (primary outcome – sensitivity analysis A: aOR 3·60, 95% CI, 1·91-6·79; p<0·0001; sensitivity analysis B: aOR 4·44, 95% CI, 2·29-8·61; p<0·0001). For the restricted analysis, sensitivity analyses did not result in significant differences between the two arms. Full results for primary and secondary outcomes are shown in Tables S5 and S6.

Sputum smear tests

Of 65 DOT patients and 54 VOT patients who started the trial 30 days or less after starting treatment 17 DOT patients and 13 VOT patients were coughing and could produce sputum at two months after treatment onset. Of these, three DOT and three VOT patients remained culture positive.

Treatment completion

Based on data from the London Tuberculosis Register and follow-up with clinics ascertained at the end of the study, 83/114 (72·81%) DOT arm patients and 90/122 VOT arm patients (80·4%) had completed treatment (chi-square p=0·18). There were three patients lost to follow up in the DOT arm and 5 in the VOT arm. Full treatment outcomes are shown in Table S8. Twelve month treatment outcomes were not available for all patients at the time of extracting; these will be reported when national surveillance data are finalised.

Hospitalisations and outpatient appointments

There were 16 hospital admission episodes in the DOT arm and 14 in the VOT arm. There were 233 unscheduled outpatient visits in the DOT arm and 169 in the VOT arm. In the DOT arm the majority of unscheduled appointments (140/233) were in the 58/114 patients who did not engage with DOT.

Patient satisfaction

Patient satisfaction surveys were incomplete (65 collected in those in the VOT arm) and 57 collected on those in the DOT arm. 91% of those who engaged with VOT reported being satisfied with their care vs 95% of those who engaged in DOT (Table S9).

Health-related quality of life

Responses for the five health dimensions measured in the EQ5D were mapped to Time-trade-off (TTO) utility scores (representing a quality-adjusted life year weight) using UK specific weightings. TTO values represent how good or bad a health state is based on a health scale from 1 (full health) to 0 (state equivalent to dead). We also used the values reported by patients on the visual analogue scale, which ranges from 1 (best health you can imagine) to 0 (worst health you can imagine). Results at baseline, 2 months and 6 months are summarized in Tables S10 and S11.

Participant resource use

Time and costs of observation for patients are shown in Tables S12 and S13. From reports at two months, DOT patients spent a mean of 29·4 (standard deviation 48.2) minutes on treatment observation per week (travel to and from appointments; waiting in clinic and appointment). VOT patients spent a mean of 1·80 (standard deviation 2·20) minutes setting up and filming videos, although time to submit videos was varied and dependent on mobile phone signal. Few patients on either arm reported lost income or missing time at work as a result of treatment observation (Table S14).

Observer resource use

Time and costs of observation and reengaging patients are shown in Tables S15-S17. From reports at two months, mean time spent observing a VOT dose was 3·2 (standard deviation 0·5) minutes; for clinic-based DOT it was 15·0 (standard deviation 12·0) minutes, and for community-based DOT (including travel time) it was 56·1

(standard deviation 53·9) minutes. The NHS staff band of the nurse observing VOT was 7; for DOT it was 6 or 7. Non-NHS community-based DOT observers included hostel staff, pharmacists, and keyworkers.

Costs

The monthly cost per patient of DOT depends on the frequency of observation, but the minimum of 3 observations per week costs £2,850 per patient for 5 months of treatment, £3,420 for 6 months, and £3,990 for 7 months. The per-patient cost is not affected by the number of patients.

In contrast, the per-patient cost of VOT depends upon the number of patients, due to the fixed costs of setting up the service, and the fixed monthly cost of the band 7 nurse to manage the service. If only 10 patients were managed by VOT then the cost per patient would be higher than if DOT 3 times per week were used, although it would still be cheaper than DOT 5 times per week (except for the longest treatment durations) and VOT provides 7 observations per week. If 25 patients were managed by the VOT service than it would be cheaper than DOT 3 times per week, and with greater numbers of patients the cost per patient of VOT falls substantially. Costs per patient for DOT and VOT for different durations of treatment are shown in Table S18.

TABLE S1: Analyses conducted.

**Study outcomes**	Primary outcome (binary)<br>≥80% scheduled observations successfully completed in the two months following enrollment		Main secondary outcome (continuous)<br>Proportion of scheduled treatment observations successfully completed in the two months following enrollment and through treatment
**Analysis strategy**	Intention-to-treat<br>Patients classified according to arm to which they were originally allocated		Restricted<br>Excluding patients with less than one week of observation in allocated arm.
**Successful observations**	Main<br>DOT: All medicines observed<br><br>VOT: All medicines observed; received but corrupted video clips	Sensitivity A<br>DOT: All medicines observed<br><br>VOT: All medicines observed	Sensitivity B<br>DOT: Some or all medicines observed; reported self-administered therapy<br><br>VOT: Some or all medicines observed; received but corrupted video clips; other technical issues with clips

TABLE S2: Costs of VOT.

Component	Cost	Source
IT set-up: one-off cost	£2,000	This study
Cloud data storage, software licences, maintenance: monthly cost	£3,270	This study
Band 7 nurse to manage the service: monthly cost	£4,425	Agenda for Change pay scale
Mobile phone: one-off cost per patient	£49	This study
Data charges and insurance for mobile phone: monthly cost per patient	£26	This study
Band 5 nurse to observe videos: monthly cost per patient	£79	This study, Agenda for Change pay scale

TABLE S3: Characteristics of eligible patients approached to take part in the trial who were recruited or who refused.

Characteristic	Recruited N (%)	Refused N (%)
Total	226	127
Age group (years)		
16-34	125 (55.3)	55 (43.3)
35-54	80 (35.4)	46 (36.2)
55+	21 (9.3)	20 (15.8)
Sex		
Male	165 (73.0)	76 (59.8)
Female	61 (27.0)	50 (39.4)
Previous TB		
No	167 (73.9)	98 (87.4)
Yes	57 (25.2)	26 (20.5)
Any social risk factor		
No	133 (58.8)	76 (59.8)
Yes	93 (41.2)	51 (40.2)
Homeless		
No	177 (78.3)	99 (78.0)
Yes	47 (20.8)	23 (18.1)
Prison		
No	207 (91.6)	111 (87.4)
Yes	18 (8.0)	12 (9.45)
Alcohol/ drug problems		
No	168 (74.3)	88 (69.3)
Yes	54 (23.9)	35 (27.6)

TABLE S4: Observation at two months (total doses observed).

	DOT		VOT	
	**N doses observed**	**%**	**N doses observed**	**%**
**ITT**				
Total scheduled	3922		6474	
Main	1774	45·2	5091	78·6
Sensitivity A	1774	45·2	4756	73·5
Sensitivity B	2300	58·6	5350	82·6
**Restricted**				
Total scheduled	2418		5893	
Main	1774	73·4	5091	86·4
Sensitivity A	1774	73·4	4756	80·7
Sensitivity B	2300	95·1	5350	90·8

TABLE S5: Observation at two months (primary outcome).

	N (%) with primary outcome		Unadjusted		Partially-adjusted		Fully-adjusted	
	**DOT**	**VOT**	**OR (95% CI)**	**p**	**aOR † (95% CI)**	**p**	**aOR † (95% CI)**	**p**
**ITT**								
Total	114	112						
Main‡	35 (30·7)	78 (69·6)	5·18 (2·94-9·12)	<0·0001	5·48 (3·10-9·68)	<0·0001	-	-
Sensitivity A	35 (30·7)	68 (60·7)	3·49 (2·01-6·04)	<0·0001	3·60 (1.91-6·79)	<0·0001	-	-
Sensitivity B	49 (43·0)	85 (75·9)	4·18 (2.36-7.38)	<0·0001	4·44 (2·29-8·61)	<0·0001	-	-
**Restricted§**								
Total	56	101						
Main	35 (62·5)	78 (77·2)	2·03 (1·00-4·15)	0·051	2·23 (1·16-4·27)	0·016	2·52 (1·17-5·47)	0·019
Sensitivity A	35 (62·5)	68 (67·3)	1·24 (0·62-2·45)	0·542	1·29 (0·71-2·34)	0·398	1·44 (0·75-2·75)	0·273
Sensitivity B	49 (87·5)	85 (84·2)	0·76 (0·29-1·97)	0.571	0·831 (0·28-2·45)	0·737	0·84 (0·28-2·46)	0·744

* Number of patients who had ≥ 80% observations successfully completed in the first two months following randomisation (the primary outcome).

† Partially-adjusted models adjusted for time since start of treatment, age, sex and treatment. Fully-adjusted models (for restricted analysis only) additionally adjusted for current social risk factor (homelessness, imprisonment, drug use, alcohol problems, immigration concern), ever lost to follow up, no recourse to public funds, mental health problems.

‡Main analysis: VOT treatment observations were classified as successfully completed if ingestion of all medicines was observed, or if video clips were received but not viewable due to a technical complication. Sensitivity analysis A: only videos for which ingestion of all medicines was observed classified as successfully completed. Sensitivity analysis B: any technical issues with VOT clips as successfully completed; and self-administered treatment as successfully completed for DOT.

§Restricted analysis included only patients who engaged initially (had at least one week of observation) on the allocated treatment arm.

TABLE S6: Observation at two months (secondary outcome).

	DOT Proportion doses observed		VOT Proportion doses observed		Mean difference in proportion doses observed (95% CI)*	p
	**Mean**	**sd**	**Mean**	**sd**		
**ITT**						
Main	0·36	0·41	0·78	0·31	0·41 (0·29-0·53)	<0·0001
Sensitivity A	0·36	0·41	0·73	0·31	0·36 (0·24-0·48)	<0·0001
Sensitivity B	0·46	0·48	0·82	0·31	0·35 (0·21-0·50)	<0·0001
**Restricted**						
Main	0·73	0·27	0·86	0·17	0·14 (0·07-0·20)	<0·0001
Sensitivity A	0·74	0·27	0·81	0·20	0·082 (0·015-0·15)	0·018
Sensitivity B	0·94	0·12	0·91	0·15	-0·026 (-0·089-0·0058)	0·103

sd, standard deviation; CI, confidence interval

*Linear regression coefficient ITT adjusted for time since start of treatment, age, gender; Restricted additionally adjusted for current social risk factor, ever lost to follow up, no recourse to public funds, mental health problems. Distributions of outcomes showed some departures from normality. As a sensitivity analysis, we therefore also tested the difference in groups using the nonparametric Wilcoxon rank-sum test, which produced similar effects, suggesting that the results were robust to this assumption.

TABLE S7: Trial outcome of DOT by DOT location.

Location	Number of patients (N=114)	Primary outcome (≥ 80% of scheduled observations completed) N (%)
Clinic	20	10 (50·0)
Community	9	6 (66·7)
Home	27	19 (70·4)
No initial engagement (less than one week on allocated intervention)	58	0

Chi-square test for difference in proportions (clinic vs community vs home – excluding never started) P = 0.348

TABLE S8: Treatment outcome by trial arm.

Outcome*	DOT		VOT	
	N	% (of 114)	N	% (of 112)
Completed	83	72·81	90	80·4
Currently on treatment	11	9·65	8	7·14
Died – TB	0	0	0	0
Lost to follow up	3	2·63	5	4·46
Transferred out	5	4·39	2	1·79
Treatment stopped/ interrupted/ not started	9	7·89	7	6·25
Withdrawn from study – no data collection	3	2·63	0	0

*Treatment outcome extracted from patient records March 2017

TABLE S9: Patient satisfaction by trial arm.

Satisfaction*	DOT (% of those with answer)	VOT (% of those with answer)
Strongly agree	30 (52·6)	29 (44·6)
Agree	24 (42·1)	30 (46·2)
Neither agree nor disagree	1 (1·8)	1 (1·5)
Disagree	2 (3·5)	2 (3·1)
Strongly disagree	0	3 (4·6)

*How much do you agree or disagree with the following statement: “I am satisfied with the way my treatment is observed”?

TABLE S10: Health-related quality of life for patients on DOT and VOT, measured by time trade-off (TTO) derived from EQ5D-3L using UK value set

	DOT			VOT		
	**n**	**Mean**	**95% CI**	**n**	**Mean**	**95% CI**
**Baseline**	102	0·74	(0·67 to 0.81)	106	0·75	(0·70 to 0·81)
**2-month follow-up**	25	0·76	(0·65 to 0·86)	47	0·75	(0·66 to 0·83)
**End of treatment**	17	0·70	(0·51 to 0·89)	23	0·73	(0·58 to 0·88)

TABLE S11: Health-related quality of life for patients on DOT and VOT, measured by EQ5D-3L visual analogue scale

	DOT			VOT		
	**n**	**Mean**	**95% CI**	**n**	**Mean**	**95% CI**
**Baseline**	73	65·2	(60·1 to 70·3)	87	67·0	(62·4 to 71·6)
**2-month follow-up**	22	73·3	(63·7 to 82·9)	42	71·4	(63·6 to 79·2)
**End of treatment**	14	73·6	(61·6 to 85·6)	23	73·7	(63·0 to 84·4)

TABLE S12: DOT participant resource use, by survey time*

	2 months				6 months			
	**N answered**	**Mean**	**Sd**	**Range**	**N answered**	**Mean**	**Sd**	**Range**
**Travel to appointment (minutes)**	12	29·3	20·4	5-60	13	28·1	34·3	0-130
**Wait (minutes)**	12	18·1	17·1	0-60	13	4·9	8·8	0-30
**Appointment (minutes)**	15	17·8	15·6	7-60	17	18·1	12·6	2-45
**Travel home from appointment (minutes)**	12	33.1	23.1	5-75	12	35.8	27.7	7-105
**Cost of travel (GBP)**	8	6·6	5·9	1·5-20	8	2·6	3·1	0-8

*estimated over the previous week

TABLE S13: VOT participant resource use, by survey time*

	2 months				6 months			
	**N answered**	**Mean**	**Sd**	**Range**	**N answered**	**Mean**	**Sd**	**Range**
**Setup (minutes)**	44	1·9	2·0	0-7	34	2·1	2·2	10sec-10min
**Film (minutes)**	44	1·0	0·9	10sec-5min	33	2·4	6·3	20sec-5min
**Submit video (minutes)**	8	Immediate to a couple of days, depending on signal			33	3·9	11·9	0-60

*estimated over the previous week

TABLE S14: Lost income and missed time from paid/ unpaid work, by survey time and trial arm*

	DOT				VOT			
	2 months		6 months		2 months		6 months	
	N answered	n (%) yes	N answered	n (%) Yes	N answered	n (%) Yes	N answered	n (%) Yes
**Lost income as a result of treatment observation (GBP)**	13	1 (7·69)	18	1 (5·56)	34	4 (11·76)	17	0 (0)
**Missed time from paid/ unpaid work as a result of treatment observation (minutes)**	12	3 (2·50)	15	1 (6·67)	30	1 (3·33)	18	0 (0)

*estimated over the previous week

TABLE S15: Clinic-based DOT observer resource use, by survey time

	2 months				6 months			
	**N answered**	**Mean**	**Sd**	**Range**	**N answered**	**Mean**	**Sd**	**Range**
**Average time observing each dose over previous week**	19	15·0	12·0	5-60	12	54·9	101·8	5-301
**Travel costs to meet with patients for re-engagement (gbp) over previous two months**	1	22·0	-	-	2	28·00	22·63	12-44
**Time spent re-engaging patients over previous two months**	6	115·83	98·81	30-270	3	115·00	85·29	45-210

TABLE S16: Community-based DOT observer resource use, by survey time

	**2 months**				**6 months**			
	N answered	Mean	**Sd**	**Range**	**N answered**	**Mean**	**Sd**	**Range**
**Average time observing each dose over previous week (including travel time)**	36	56·1	53·9	2-275	19	46·5	30·4	5-110
**Travel costs to meet with patients for re-engagement (gbp) over previous two months**	8	49·5	40·52	3-116	1	4	-	-
**Time spent re-engaging patients over previous two months**	13	187·69	182·71	12-630	3	244·00	244·40	72-480

TABLE S17: VOT observer resource use, by survey time

	2 months				6 months			
	**N answered**	**Mean**	**Sd**	**Range**	**N answered**	**Mean**	**Sd**	**Range**
**Average time observing each dose over previous week**	95	3·2	0·5	1·7-5·1	69	9·7	44·1	0·5-364
**Travel costs to meet with patients for re-engagement over previous two months**	66	6·66	4·20	1·68-22·0	10	5·64	1·78	3·20-9·20
**Time spent re-engaging patients over previous two months**	81	135·9	151·3	3-960	42	57·4	76·5	3-280

TABLE S18: Costs per patient of DOT and VOT, by treatment duration.

	Cost per patient (£)						
**Duration of treatment (months)**	**5**	**6**	**7**	**8**	**12**	**15***	**24***
DOT							
3 obs. per week	2,850	3,420	3,990	4,560	6,840	8,490	13,440
5 obs. per week	4,750	5,700	6,650	7,600	11,400	13,050	18,000
VOT (incl. set-up)							
10 patients	4,620	5,500	6,370	7,245	10,745	13,280	20,875
25 patients	2,195	2,610	3,020	3,435	5,085	6,280	9,870
50 patients	1,385	1,645	1,900	2,160	3,200	3,950	6,200
100 patients	980	1,160	1,345	1,525	2,255	2,780	4,365
200 patients	780	920	1,065	1,210	1,785	2,200	3,445
VOT (excl. set-up)							
10 patients	4,420	5,300	6,170	7,045	10,545	13,080	20,675
25 patients	2,115	2,530	2,940	3,355	5,005	6,200	9,790
50 patients	1,345	1,605	1,860	2,120	3,160	3,910	6,160
100 patients	960	1,140	1,325	1,510	2,235	2,760	4,345
200 patients	770	910	1,055	1,200	1,775	2,190	3,435

*For durations >12months, costs falling in the second year are discounted at 3.5%

FIGURE S1: Proportion of clips with technical issues during the study.

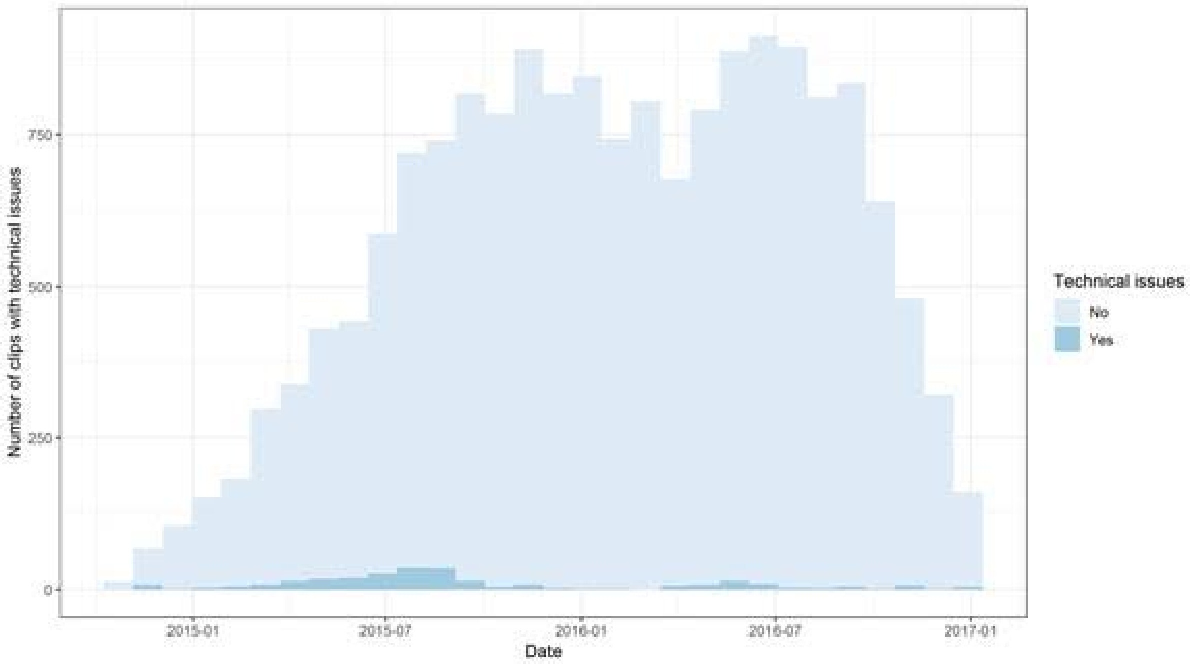

FIGURE S2: Distribution of number of clips with technical issues by patient.

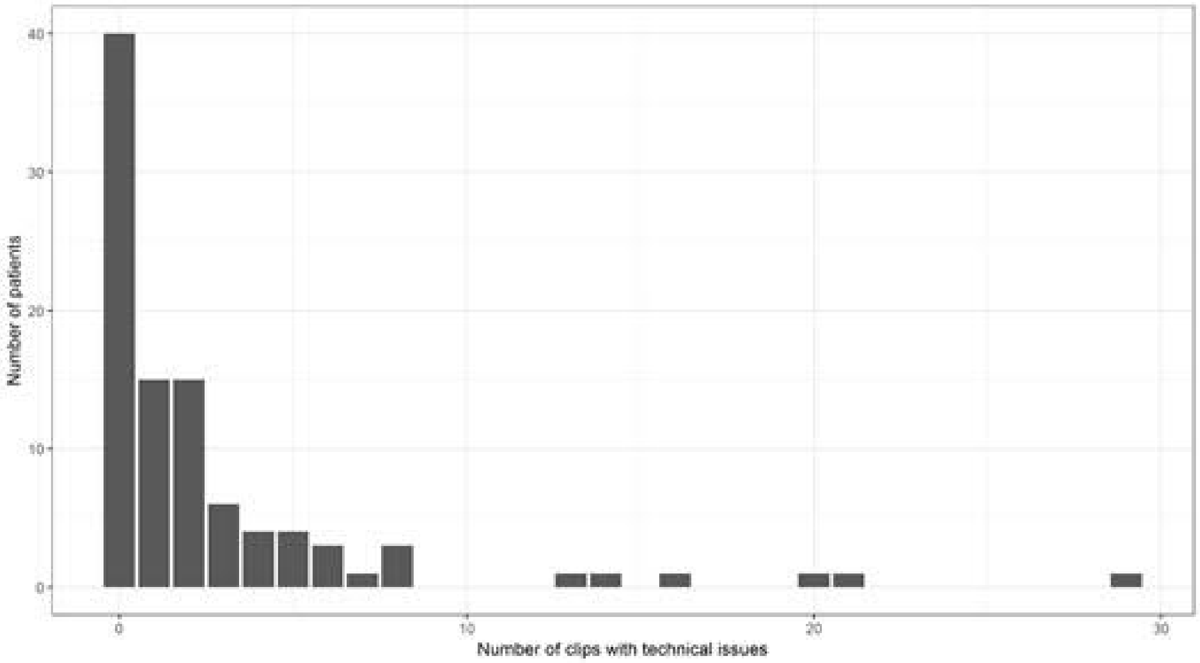

Virtually Observed Therapy (VOT)

RCT

Andrew C Hayward – Chief Investigator

2/28/2014

VOT RCT - Standard Protocol Items: Recommendations for Intervention Trials (SPIRIT) Checklist

Contents

Administrative information 29

Introduction 31

Methods: Participants, interventions, and outcomes 32

Methods: Assignment of interventions (for controlled trials) 42

Methods: Data collection, management, and analysis 43

Monitoring 48

Ethics and dissemination 49

Appendices 51

Administrative information

1) Title: A multicentre, analyst-blinded, randomised, superiority study to compare the efficacy of Video Observed Treatment (VOT) versus Directly Observed Treatment (DOT) in supporting adherence in patients with active tuberculosis.

2a) Trial identifier and registry name: TBC

2b) All items from trial registration dataset

3) Protocol Version: 28/01/2014

4) Funding: National Institute of Health Research (NIHR) Programme Grant

5) Roles and Responsibilities

5a) Names Affiliations and roles of protocol contributors

Dr Andrew Hayward, UCL Centre for Infectious Disease Epidemiology

Chief Investigator

Professor Ibrahim Abubakar, UCL Centre for Infectious Disease Epidemiology –

Co-lead & Trials advisor

Dr Alistair Story, University College London Hospitals, Advice VOT implementation and TB services liaison

Dr Marc Lipman – UCL, North Central London London TB Service – Clinical advisor

Dr Rob Aldridge – UCL Centre for Infectious Disease Epidemiology – Statistical Advisor

Dr Tim McHugh – UCL, Department of Infection and Immunity - Microbiology Advisor

Dr Peter White – Imperial College London – Health Economics and Modelling Lead

AH and AS conceived the study, IA, ML, RA, TM contributed to study design, RA is conducting the primary statistical analysis. PW will lead the economic analysis.

5b) Trial Sponsor:

University College London

UCL Sponsor Representative - David Wilson; david.wilson@ucl.ac.uk

5c) Role of Study Sponsor and Funders

The study sponsor has responsibility for the overall conduct and quality of the trial. The funder influenced study design through the peer review process but otherwise the funder and sponsor had and will have no role in the collection, management, analysis, and interpretation of data; writing of the report; and the decision to submit the report for publication.

5d) Additional roles and responsibilities.

Principal investigator (PI)

- Design and conduct of TB Reach VOT trial
- Preparation of protocol and revisions
- Preparation of investigators brochure (IB) and CRFs [case report forms]
- Organising steering committee meetings
- Publication of study reports
- Determining members of TMC [Trial Management Committee]

Steering committee (SC)

- (see section 5a for members) plus Study co-ordinator (Elizabeth Garber)
- Agreement of final protocol
- Liaising with study sites
- Arranging site quality control visits
- Reviewing progress of study and if necessary agreeing changes to the protocol and/or investigators brochure to facilitate the smooth running of the study.
- Budget administration and contractual issues with individual centres

External Trials Management Committee (TMC)

- Chair – Professor Andrew Nunn –UCL MRC Clinical Trials Unit
- Members – Professor Mark Woodhead, Consultant in Respiratory and General Medicine, Central Manchester University Hospitals, Dr Ann Chapman, Consultant in Infectious Diseases – Monklands Hospital, Airdrie, Scotland, Josie Mavromatis – Lay Representative
- Advising on study design
- External scrutiny of study procedures, implementation and recruitment
- Review of SUSAR [Serious unexpected suspected adverse events]

Data monitoring Committee

The study is a non-pharmaceutical intervention with no planned stopping rules or interim analyses. There will therefore be no formal data monitoring committee. However the trial statistician (Rob Aldridge) will prepare data reports for the TMC to be checked by an independent statistician from MRC CTU.

Data manager

- Maintenance of trial IT system and data entry
- Data verification
- Responsible for trial master file

Lead investigators

In each participating centre a lead investigator (senior nurse or clinician) will be identified, to be responsible for identification, recruitment, data collection and completion of CRFs, along with follow up of study patients and adherence to study protocol and investigators brochure. .

Introduction

6a & b) Background

In the United Kingdom, tuberculosis patients at high risk of poor adherence to tuberculosis treatment are recommended to have directly observed treatment (DOT) to minimise the risk of relapse, drug resistance and spread of infection. Groups eligible for DOT include patients with social risk factors (including alcohol or drug use, history of imprisonment, homelessness), mental health problems, evidence of poor adherence, previous TB treatment and clinically complex disease requiring extra support. Direct observation can be time and resource intensive for both patients and NHS services, requiring at least three visits per week. The three times weekly regime used for DOT may also be less satisfactory than a daily regime. In the UK, surveillance data suggest that a high proportion of patients who are recommended for DOT do not receive DOT. In the US DOT is the recommended mode of treatment for all TB patients. Recently the University of San Diego has developed a smart phone “app” allowing patients to easily submit video recording of themselves taking treatment to a secure server for reading by a health care worker (Video Observed Treatment – VOT). This has been shown to be effective and highly acceptable in non-socially complex cases in the US but has not been trialled in more socially complex patients such as those recommended for DOT in the UK. Our study team have pioneered the use of VOT with the pan London Find&Treat TB outreach service (but without the use of a dedicated smart phone app) in socially complex cases in London and again found it to be highly acceptable to patients. We have also modelled potential cost savings through using DOT rather than VOT

and found VOT to be considerably cheaper to deliver than DOT. We are collaborating with the University of San Diego to use their VOT “app” in a trial of effectiveness in UK patients eligible for DOT.

7) Research Hypothesis: In comparison to DOT, VOT increases the proportion of patients who have more than 80% of doses observed during a 2 months period.

Primary Objective: To assess the effectiveness of VOT in comparison to DOT on adherence to treatment

Additional Objectives

- To measure the impact on adherence over 6 months
- To measure impact on loss to follow up and treatment completion
- To measure effect on culture conversion and development of resistance
- To measure impact on transmission
- To measure impact on quality of life and patient satisfaction
- To assess cost effectiveness of VOT

8) Description of trial design.
The TB Reach VOT trial is designed as a randomised, controlled, analyst blinded multicenter superiority trial with two parallel groups (VOT and DOT) with a 1:1 allocation and a primary endpoint of 80% of doses being observed over a 2 month period .

Methods: Participants, interventions, and outcomes

9) Description of study settings : Tuberculosis outpatient clinics in London and Birmingham and the London TB outreach service: Find&Treat.

10) Inclusion criteria: Any patient 16 years of age or older eligible for DOT at participating clinics.

Exclusion criteria

1) Patients who are eligible for DOT but not suitable for VOT due to:

a) Need for injectable treatment regime
b) No access to the facilities to charge a smart phone.

2) Patients in whom the primary outcome cannot be measured because they have less than 2 months of treatment remaining.

3) MDRTB patients requiring twice daily treatment who will be recruited into a non randomised arm of the study where VOT is offered, with the same follow up as the other study arms. This represents small numbers of patients and is planned because DOT is highly difficult to organise in this group and VOT is therefore already considered the optimal arrangement.

11a) Interventions for each group

VOT: Daily submission of VOT clip using dedicated smartphone with pre-loaded app allowing upload to a secure server. Participants will be trained in how to lay out each drug on a labelled laminated medication sheet with a space for each drug and take each drug individually saying either the name of the drug or the colour of the pills, size, and the number taken. Participants will be asked to show their mouth is empty by opening their mouth and sticking out their tongue and finally be asked to report any symptoms from a list of key side effects (which will be printed on the reverse of the laminated medication sheet – Appendix 1 & 2). Training will include submission of test videos.
VOT clips will be submitted automatically as soon as the phone is connected to a cellular data network (data plan provided with phone) or wireless network.
VOT clips will be read by a study nurse/VOT observer daily during weekdays with weekend clips read on Mondays.
No incentives or travel costs will be provided but participants will be able to make use of study smartphone for e-mails, domestic telephone calls and internet searches (limited data downloads apply).
After any missed dose of VOT the observer should attempt to contact the patient by telephone to find out what the problem is and encourage submission of further video clips. If this is a technical problem that cannot be resolved over the phone a visit will be arranged to resolve this. If they are unable to contact them within 24 hours of a missed dose they will contact the case manager to discuss.

DOT: A trained health professional, or responsible lay person supported by a trained health professional, provides the prescribed medication and observes the patient swallowing every dose (or for some schedules observing doses during weekdays with self administered therapy at weekends). Organised by clinic according to usual practice – may be

a) clinic based
b) community based working with a responsible professional such as a hostel worker or pharmacist
c) through a DOT worker outreaching DOT.

Clinics may choose to use incentives and provide travel costs as per normal practice

After each missed dose of DOT the case manager should attempt to contact the patient by telephone to find out what the problem is and encourage re-engagement.

11b) Criteria for discontinuing or modifying allocated interventions for a given trial participant.

If participants have repeated episodes of non-adherence in either DOT or VOT arms they will crossover to the other arm of the trial.

Non adherence is defined as follows:
Patients on daily therapy are considered non-adherent after missing three daily doses within one week or two doses per week in two consecutive weeks.
Patients on three times per week therapy are considered non-adherent after two or more doses are missed within two weeks.
(Taken from - Tuberculosis case management and cohort review – Guidance for health professionals. Royal College of Nursing).

If, a VOT arm participant meets the above definition of non adherence, the VOT observer will arrange face-to-face re-training to re-engage the participant with VOT. If retraining cannot be arranged they will be referred back to the clinic for DOT and enter the DOT arm of the study. If the patient agrees to retraining, but has a subsequent episode of non-adherence (see definition above) they will be referred back to the clinic for DOT and enter the DOT arm of the study.

If a DOT arm participant meets the above definition of non-adherence, the case manager will arrange a face-to-face meeting to re-engage the participant with DOT. If this does not lead to agreement to recommence DOT the participant will be offered to swap to the VOT arm of the trial. If the patient does agree to attend DOT but has a subsequent episode of non-adherence (see definition above) they will be offered VOT and enter the VOT arm of the study.

Changeover of study arm will only be made after a case discussion between a senior member of the study team (AH, AS or ML and the clinic case manager) to review the evidence that they meet the criteria above.

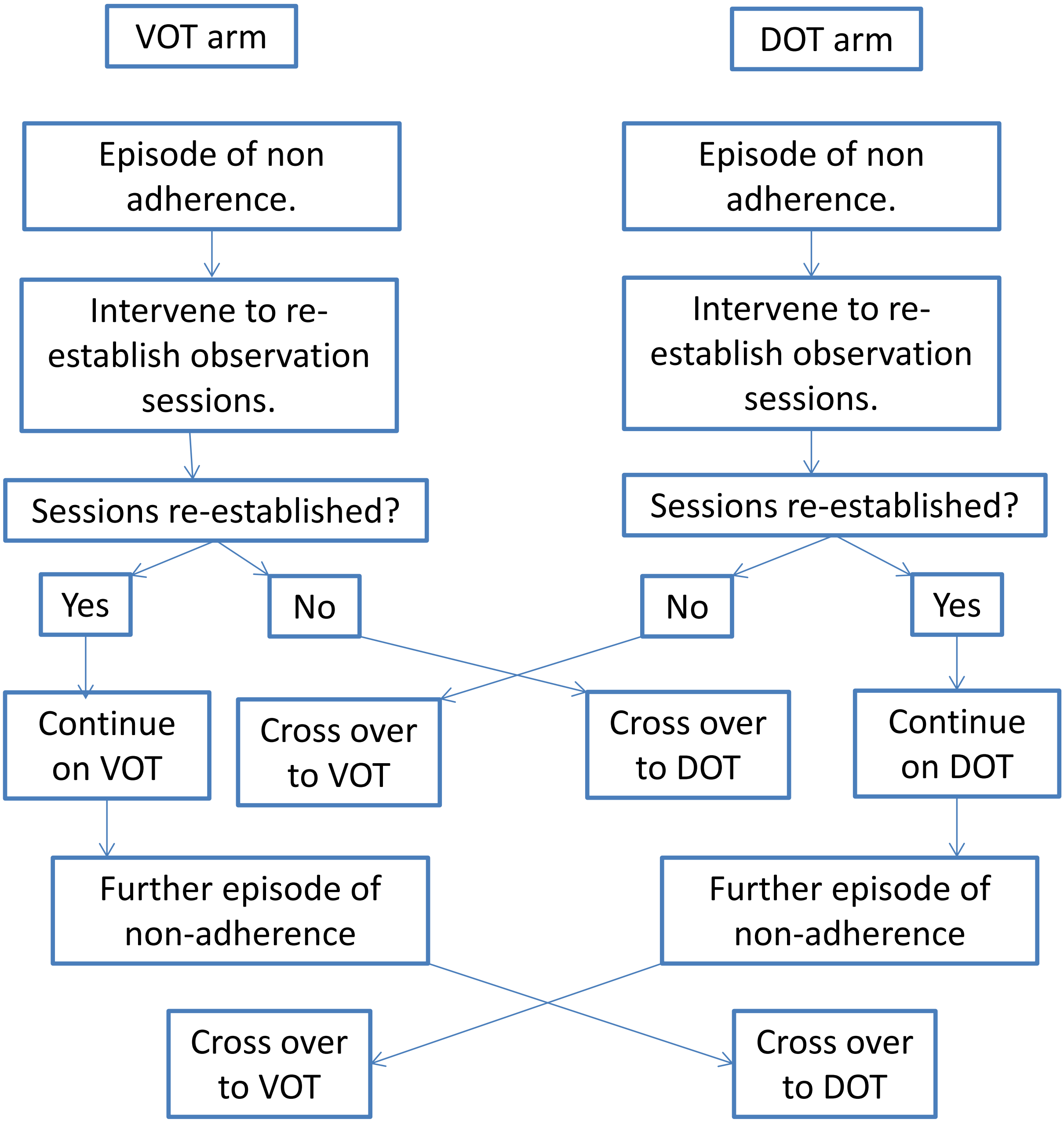

Patients switching from VOT to DOT during the first 2 months of follow up will be considered as VOT failures for the primary outcome. Patients switching from DOT to VOT during the first 2 months of follow up will be considered as DOT failures for the primary outcome.

For patients who have already crossed over study arms and continue to be non-adherent the clinic responsible for their care will determine the preferred mode of continuing to support adherence for the remainder of their care.

11c) Strategies to improve adherence to intervention protocols, and any procedures for monitoring adherence (e.g, drug tablet return, laboratory tests)

Participants will receive training in how to submit VOT clips during a household or clinic visit (according to participant preference) with additional training provided if participants fail to return satisfactory clips.

Adherence is primary outcome – see section 12.

11d) Relevant concomitant care and interventions that are permitted or prohibited during the trial.

In the DOT arm clinics may use incentives and enablers (mainly travel expenses) as per usual practice.
VOT arm patients are able to make use of study smartphone for e-mails, domestic telephone calls and internet searches (limited data downloads apply). However on enrolment they will be asked to sign a form (Mobile Phone Sign Out Form – Appendix 3) agreeing to the appropriate use of the mobile phone and the understanding that they may be returned to the DOT arm if the phone is lost or stolen. A copy will be retained by the study and the participant. Similarly, a release form (Mobile Phone Return Form – Appendix 4) will be completed and a copy given to the patient when the patient returns the phone to a team member.

Dosset boxes are allowed in either arm.
VOT arm participants living outside of mobile phone signal coverage areas will receive help in identifying a local wireless network they can log onto.
VOT observers may contact participant via email, text or telephone to encourage them to continue to submit.
Patients in VOT or DOT arms may be referred to relevant agencies to address social /addiction issues. The responsibility for this referral rests with the treating clinic.

12) Outcomes

Primary Outcome Measure:

Proportion of participants having more than 80% of scheduled VOT/DOT sessions successfully competed in the 2 months following randomisation (binary aggregation) in participants with a minimum of 6 weeks follow up data.

Secondary Outcome measures:

Collected continuously:

- Proportion of doses observed over 2 months (continuous variable – comparison of means)
- Proportion of doses observed over 6 months (continuous variable – comparison of means)
- Reported side effects

Collected directly by study at baseline and through telephone interview of participant at 2 and 6 months:

- Quality of Life (EQ5D)
- Participant satisfaction (Likert scale)
- Participant resource use for DOT/VOT (Participant time, missed work)
- Employment
- Hospitalisation

Collected directly by study through questionnaire for VOT/DOT observer at 2 and 6 months:

- Time spent observing each dose over the previous week (including travel time where relevant) – staff grade and employer.
- Time spent re-engaging patients with DOT/VOT.
- Any major side effects requiring a change in treatment regime.

Collected by treating clinic - Sputum smear conversion at 2 months

Collected from national surveillance data

- Treatment outcome at 12 months (Completed, loss to follow up, transferred out, died)
- Acquisition of new resistance
- Membership of a transmission cluster (based on data from national strain typing service)

13) Participant timeline

In patients who are eligible for DOT there is concern about adherence and potential loss to follow up. In a clinic setting DOT is therefore generally set up and arranged during the clinic appointment during which it is first discussed. We wish to mirror this rapid service in the trial and therefore will aim to complete recruitment during the clinic session at which DOT is first discussed with the patient. This will also minimise loss to follow up prior to being able to establish the intervention.

In order to meet the needs of participating clinics and allow this rapid recruitment over multiple sites there are three options for recruitment:

A) When clinics first join the study, study nurses will attend outpatient clinics and be able to recruit patients referred by the clinic nurse through a face to face meeting. Clinic nurses will be encouraged to observe as part of their training in recruitment. Study nurses may also attend clinic if they are given advanced warning that a patient who is eligible for DOT (or already on DOT) will be at clinic.
B) When study nurses are not available on site, and clinic resources permit, clinic nurses will: explain the study; provide study information; seek consent; randomise the patient; complete the baseline questionnaire with the patient (largely populated from existing data collected during the course of routine care) and liaise with the study team to organise follow up.
C) When study nurses are not available on site and there are insufficient clinic resources to recruit patients directly, the clinic nurse will arrange a video conference with the study team who will: discuss the study with the patient; seek written consent; randomise and arrange onward follow up. The clinic nurse will complete the baseline questionnaire (largely populated from existing data collected during the course of routine care).

Day zero. Time zero – Clinic identifies patient eligible for DOT during outpatient or inpatient episode. (A, B, C)

Day zero. Time 0-5 mins – Clinic nurse discusses the recommendation that the patient be treated with DOT and the possibility of being involved in a study of how best to observe treatment (face to face) or using video observed therapy. (A, B, C)

Day zero – Time 5-25 minutes - Clinic nurse provides patient information sheet to patient and allows them to assess whether they wish to receive further information. (A, B, C)

Day zero – Time 25-50 minutes –Clinic nurse (A) or study nurse (B or C) explain the study, seek written consent, randomise and arrange onward care.

Day zero – Time 50-60 minutes - study nurse (A) or clinic nurse (B, C) collect baseline data largely populated from existing clinical data/care records.

Day zero Time 60 minutes -120 minutes (A & B) – Training in use of VOT and provision of smart phone.

Day one (or first working day after day zero) – (C) VOT training up to 1 hour at patients home or at clinic depending on patient preference.

Day one (A & B) or two (C) – VOT arm – patient attempt to submit VOT clip unaided, study team liaises and arranges further training if necessary.

Day one (or first scheduled DOT appointment) – patient begins attending for DOT.

0-2 months – regular DOT/VOT with adherence data collection and monitoring of side effects.

2 months – Study team contact participant by telephone to complete follow up questionnaire covering satisfaction with DOT or VOT, Time spent on DOT or VOT, Employment and quality of life, periods of time spent away from home.

Regular out-patient appointment closest to 2 months clinic nurse requests sputum sample to test for culture conversion at local laboratory.

6 months - Study team contact participant by telephone to complete end of follow up questionnaire covering satisfaction with DOT or VOT, Time spent on DOT or VOT, Employment and quality of life, periods of time spent away from home. For patients still requiring onward care the study will liaise with the clinic and Find&Treat to organise continuing DOT or VOT.

Schematic of participant timeline

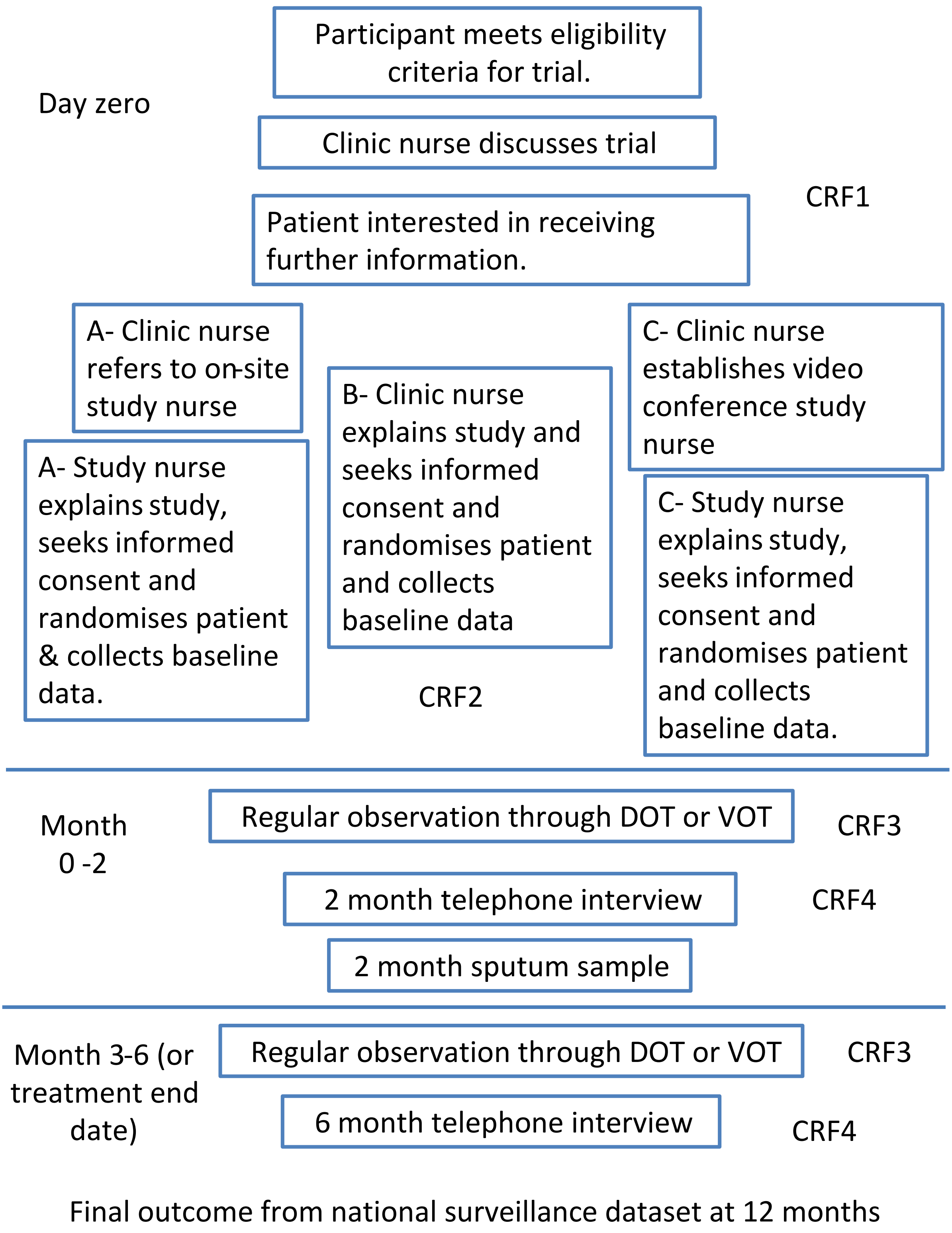

14) Sample size:

A widely used programmatic measure of acceptable adherence is that patients are known to have taken at least 80% of their scheduled doses. This is particularly important early in treatment. We therefore define an adherent patient as one who is observed to take over 80% of their treatment and will compare the proportion of patients who are adherent in each arm over the first 2 months from randomisation as the primary outcome.

We have examined the power implications of a range of realistic differences in the primary outcome with 90% power at the 5% significance level (2 sided) with equal numbers in intervention and control arms of a superiority trial (table 1).

We have reviewed adherence data from Cohort review of TB patients and the VOT pilot and this suggests the 3rd or 4th scenarios are realistic.

Table 1 – Sample size needed at differing estimates of adherence and effect size.

	**50% vs 70%**	**40% vs 60%**	**65% vs 80%**	**60% vs 75%**
**Intervention**	**121**	**126**	**181**	**200**
**Control**	**121**	**126**	**181**	**200**
**Total**	**242**	**252**	**362**	**400**

We will aim to recruit 400 participants into the trial over a two year period.

15) Recruitment Strategies for achieving adequate participant enrolment to reach target sample size.

Recruitment relies on conducting the study in multiple centres. We have focussed recruitment of clinics based on existing good relationships and high numbers of cases eligible for DOT. We are also approaching Birmingham clinics to increase numbers.

Based on the following assumptions we expect to be able to recruit 400 patients over a 2 year period.

Assumptions:

The annual number of eligible patients at each clinic will be similar to the annual number of patients reported to national enhanced surveillance in 2010 and 2011 as having social risk factors (homelessness, problem drug or alcohol use or history of imprisonment) but not on DOT, plus the number of patients already on DOT. This averaged at 420 eligible per annum.

It was also assumed that:
95% of those eligible for DOT are also eligible for the trial
Clinics attempt to recruit 75% of those eligible
2/3 of those who are approached agree to participate (based on pilot data VOT acceptance rates)

Note – As MDRTB patients with twice daily treatment are now not included in the randomisation we have not included MDRTB in the estimates. The true numbers of eligibles will be higher as we have ignored, previous history of TB and history of poor adherence, also this is based on incident eligible cases whereas we will also be recruiting patients who are currently on DOT.

We will initially attend outpatient appointments to induct clinics and promote the trial. We will visit clinics monthly for quality control visits.

Another strategy for achieving good recruitment is an emphasis on recruiting patients on the day the study is first introduced to them. As this is a hard – to – engage group delaying this could lead to substantial drop-out.

Methods: Assignment of interventions (for controlled trials)

Allocation:

16a) Sequence generation

We will use computer generated randomisation by minimisation to ensure balance across study sites and stage of treatment at which recruitment took place 1) at start of treatment; 2) after start but within first 2 months of treatment; 3) After first 2 months of treatment.
a) Whether or not a patient has previously demonstrated poor adherence
b) Whether or not a patient is already on DOT at the time of recruitment.

This will ensure that treatments are balanced within the four strata.

16b - Allocation concealment mechanism

Randomisation will be commissioned from SealedEnvelopeTM (http://www.sealedenvelope.com/) and conducted centrally using an integrated internet/SMS text message randomisation service. Clinics with ready access to the internet will randomise using the internet service – the remaining clinics will use the SMS/text system or telephone the study centre who will use the internet system and pass on the allocation. The systems also check for consent, study inclusion and exclusion criteria before allowing randomisation.

16c) Implementation

The allocation sequence will be generated by SealedEnvelopeTM. Participant enrolment and assignment to intervention will be done by the study nurse or clinic nurse (see Participant timeline – section 13)

17a) Blinding (masking)

It is not possible to blind participants or care providers to the intervention.
It is also not possible to blind those undertaking interviews at 2 and 6 months, as these questions include information on participant time taken to have DOT or VOT which will make the allocation obvious.
Data analysis will be blinded to the allocation for the primary outcome by ensuring the statistical files for analysis are prepared blind to the intervention.

17b) If blinded, circumstances under which unblinding is permissible – not applicable.

Methods: Data collection, management, and analysis

18a) Data collection methods

Recruitment data (CRF 1 - Appendix 5): Recruiting clinics will collect this data and retain the full recruitment log sheet which includes patient identifiers, but the anonymous portion of the

form on the characteristics of patients who were approached for the trial but declined to participate will be provided to the study team. This will include age, sex and factors that make them eligible for DOT.

Baseline Data (CRF 2 – Appendix 6): Study or clinic nurses will collect baseline data at the time of initial recruitment. This mainly consists of data already routinely collected by clinics, covering patient demographics, disease categorisation and factors that make them eligible for DOT. It will also include the validated EQ-5D quality of life scale and information on employment status and approximate weekly salary

VOT/DOT diary (CRF 3 – Appendix 7):

This will be recorded by the DOT observer at the time of the scheduled session and by the VOT observer at the time of reading scheduled video clips. DOT/VOT observers will be trained to complete (CRF 3 – see section 18a) consistently. CRF3 will be faxed/emailed on a weekly basis to the study centre with the study centre following up with DOT observers when weekly forms are not returned. CRF3 will be completed directly at the study centre on the VOT reading system.

The VOT reading system includes: fields to identify when doses are scheduled; when clips are submitted and read and the following categorisation of clips:

Patient took meds?

- Yes, all meds
- Yes, some meds
- No meds taken
- Unable to tell

This data will be used for the primary outcome and secondary adherence outcome measures.

The system also collects additional data on video and audio quality. The system collects data on geo-location at the time of recording. This will be collected if the participant provides separate consent for this.

DOT: A DOT diary will be completed by DOT observers showing when doses are scheduled to be observed, whether or not the participant attended, when they attended and whether they took the medicines categorised as above.

Patient 2 and 6 month follow up (CRF 4a & 4b – Appendix 8): The study team will contact the participant by telephone to complete a 5 minute questionnaire covering

- Quality of Life (EQ5D)
- Participant satisfaction (Likert scale)
- Participant resource use for DOT/VOT (Participant time, missed work)
- Employment
- Hospitalisation

VOT/DOT observer 2 and 6 month follow up (CRF 5a & 5b – Appendix 9): This will be completed by the VOT/DOT observer and contain the following information.

- Time spent observing each dose over the previous week (including travel time where relevant) – staff grade and employer.
- Time spent re-engaging patients with DOT/VOT.

2 month sputum sample: At the outpatient appointment closest to 2 months after randomisation the clinic nurse will request a sputum sample and submit this to the local microbiology laboratory for smear and culture.

End of VOT/DOT participant engagement (CRF 6 – Appendix 10):

Semi-structured interviews will be performed with a purposively sampled subset of DOT and VOT participants to understand issues related to provision of DOT/VOT.

12 month follow up (CRF 7 – Appendix 11): The study data manager will work with PHE to link the participant register with national surveillance data to ascertain the following routinely recorded variables.

- Treatment outcome at 12 months (Completed, loss to follow up, transferred out, died)
- Acquisition of new resistance
- Membership of a transmission cluster (based on data from national strain typing service recorded in Enhanced surveillance)

Processes to promote data quality include:

- Training of study and clinic nurses
- Training of DOT and VOT providers
- Weekly submission of DOT diaries + Weekly VOT summary report
- Chase up of DOT provider if weekly forms not submitted
- Quality control visits to clinics every 3 months
- Double data entry for all paper-based CRFs

18b Plans to promote participant retention and complete follow-up, including list of any outcome data to be collected for participants who discontinue or deviate from intervention protocols

The study is predicated on maintaining regular contact with participants to monitor adherence.

Participants who are lost to follow up but have not withdrawn from the study, scheduled observations after loss to follow up will be recorded as not having been completed. It will also remain possible to obtain their final outcome data from national tuberculosis surveillance. For participants in the DOT arm who clinics decide to step-down care from DOT to self-administered therapy data will be collected on outpatient clinic attendance and collection of medications.

19) Data management

All data entered onto paper questionnaires will be double entered into the master data base with automatic range checks where appropriate. VOT data from the VOT reading system will be held on a secure NHS compliant server which hosts the VOT system which will be backed up nightly. Weekly data integrity reports will be run on the system using automated routine. Monthly data exports will be made and data merged into the master data file. The master data file will be stored on a UCL password protected network with access restricted to study personnel. This will be backed up automatically on a daily basis. The master data file will use the unique study number allocated at the time of randomisation but will not include

personal identifier information. This identifiable information will be held in a separate password protected file on the same network drive in the form of a look up table.

Statistical methods

20a) Statistical methods for analysing primary and secondary outcomes.

Characteristics of those randomised and those not randomised due to refusal or exclusion criteria will be compared. Baseline characteristics of those randomised to intervention and control arms will be compared to check for balanced randomisation. An intention to treat analyses will be used. Categorical outcomes will compared across groups using the chi-square test or Fisher's exact test as appropriate. Continuous variables will be compared across groups using the Wilcoxon rank-sum test.

For some DOT schedules weekend doses are planned to be self-observed due to difficulty in providing DOT at the weekend. For the primary outcome these doses will not be considered in the denominator of scheduled doses, however, as this is a valuable advantage of VOT, secondary analyses will include these weekend doses as part of the treatment schedule). When participants are in hospital their dose will be considered to have been observed. When participants are in prison or custody the dose will be considered to have been observed if it can be verified with offender health that they were aware of the treatment regime. When patients are out of the country VOT participants will be encouraged to continue to take VOT clips which can either be submitted via a wifi connection or will automatically submit on return to the UK. Doses due during time abroad will not be considered as part of the primary outcome for either arm.

Patients who cross-over trial arms (see section 11b) prior to the end of 2 month follow up will be considered a failure for the primary outcome.

20b) Methods for any additional analyses (eg, subgroup and adjusted analyses)

Where there is evidence of unbalanced randomisation, secondary analysis of unbalanced variables will be adjusted for in analyses, if they are associated with outcome, using multivariable regression models as appropriate. An assessment of potential effect modifiers will be performed using interaction terms in the statistical models. If there is evidence of interaction effects will be reported in subgroup analyses.

20c) Definition of analysis population relating to protocol non-adherence (eg, as randomised analysis), and any statistical methods to handle missing data (eg, multiple imputation)

Approaches such as the sensitivity analysis described in Carpenter et al will be explored to assess whether the data are not “missing at random” before using multiple imputation to infer missing data points.
Carpenter , J., Pocock, S. and Lamm, C. J. (2002) Coping with missing data in clinical trials: a model based approach applied to asthma trials. *Statistics in Medicine*, 21, 1043-1066

Patients who cross-over trial arms (see section 11b) prior to the end of 2 month follow up will be considered a failure for the primary outcome. Data post cross over will be considered in a separate analysis comparing adherence during periods of VOT with adherence during periods of DOT.

Monitoring

21a) Data monitoring There is no formal DMC but the trail statistician will work with an independent MRC CTU statistician to prepare data reports for the DMC.

21b) Description of any interim analyses and stopping guidelines.

As this is a non-pharmaceutical behavioural intervention no interim analyses or stopping guidelines are proposed.

22) Harms

The following will considered as potential serious adverse events

- Loss to follow up.
- Death from tuberculosis.
- Breaches of data security.
- Violence to study personelle during the course of participant interaction.
- Complaints about the study from participants or participating centres.

These will be reported to the study coordinator and the study chief investigator who will investigate these, including discussion with affected patient's case managers and findings will be reported to the steering committee and data monitoring committee. Summary reports of adverse events and actions taken will be presented to the independent TSC.

23) Auditing: As part of the NHS R&D approval process the project and individual study sites may be independently audited as part of their quality control processes. No separate auditing is planned.

Ethics and dissemination

24) Research ethics approval

The study has previously been approved by the Essex Multicentre Research Ethics committee. A substantial amendment reflecting the content of this protocol will be submitted for consideration in March 2014.
Study site R&D approval will also be obtained for all recruiting sites.

25) Protocol amendments

The study steering group will agree any protocol amendments and where these are major amendments discuss them with the chair of the TSC. The project manager will ensure substantial amendments are: approved by the ethics committee and site R&D committees; reported to the trial registry and communicated to study sites. Changes in the protocol will be highlighted in supplementary appendixes of publications.

26a) Written consent will be obtained for participation in the study by study nurses or clinic nurses (section 11).

26b) Additional consent provisions for collection and use of participant data.
Participants in the VOT arm of the study will be asked whether they will allow the study access to geo-locator information from the telephone for the purpose of studying flexibility in where patients take their treatment.
Participants in the VOT arm will also be asked whether they will allow the study to keep video clips for the following purposes.

- Behavioural analysis of VOT.
- Assessment of physical correlates of response to treatment.
- Development of training materials to use with patients and staff involved in VOT or for teaching of healthcare students.
- Development of materials to be used in conference presentations.
- Development of materials to be made publically available on web sites or other media.

Separate written consent will be sought by the study nurse at the time of VOT training so the participant can indicate which (if any) of these additional data uses they agree to. Willingness to make this data available is not a pre-condition for enrolment in the main study.

27) Confidentiality

Information on potential trial participants will be collected in anonymous form.(CRF1) All other CRFs will be identified using the unique participant id allocated at randomisation. The master data file will be stored on a UCL password protected network with access restricted to study personnel. This will be backed up automatically on a daily basis. The master data file will use the unique study number allocated at the time of randomisation but will not include personal identifier information. This identifiable information will be held in a separate password protected file on the same network drive in the form of a look up table.

Study sites will also maintain separate look up tables on NHS password protected computers.

All data analyses will be conducted on anonymised data with no identifiable data leaving the study site. Analyses will be reported at a level preventing deductive disclosure.

28) Declaration of interests

Study investigators and principal investigators have no financial or other competing interests.

29) Access to data

The Data manager will oversee the intra-study data sharing process, with input from the TSC.

The principle investigator, study statistician and independent statistician will be given access to the cleaned data sets. Project data sets will be kept as described in section 19 and confidentiality will be protected as described in section 27.

Ancillary and post-trial care

30) Provisions, if any, for ancillary and post-trial care, and for compensation to those who suffer harm from trial participation

Care of patients at the end of the trial will remain the ultimate responsibility of the clinicians looking after them. Patients that are enrolled into the study are covered by indemnity for negligent harm through the standard NHS [National Health Service] Indemnity arrangements. The trial sponsor (UCL) has insurance to cover for non-negligent harm associated with the protocol.

31a) Dissemination policy

The primary outcome papers of this study will be approved by the Steering Committee as will any other analyses presented as a result of this work.

31b) Authorship eligibility guidelines and any intended use of professional writers.

The authors of VOT publications will be listed as….Disputes regarding authorship will be settled by the Principal investigator after consultation with the TMC.

31c) Plans, if any, for granting public access to the full protocol, participant-level dataset, and statistical code

No later than 3 years after the collection of the 12 month follow up data we will deliver a completely de-identified data set to an appropriate data archive for sharing purposes.

Appendices

Appendix 1 & 2:	Laminated
	Appendix 1 - Medication sheet also referred to as “Pill Form”
	Appendix 2 – List of side effects
Appendix 3:	Mobile Phone Sign Out Form –
Appendix 4:	Mobile Phone Return Form –
Appendix 5:	CRF 1 - Recruitment / Eligibility data
Appendix 6:	CRF 2 - Baseline Form & EQ5D
Appendix 7:	CRF 3 - VOT / DOT diary

Appendix 8: CRF 4a & 4b - 2 month versus 6 month participant follow-up survey

Appendix 9: CRF 5a & 5b - 2 month versus 6 month VOT/ DOT observer survey

Appendix 10: CRF 6 - End of VOT/DOT participant engagement

Appendix 11: CRF 7 - 12 month follow-up

Informed consent materials

32) Model consent form and other related documentation given to participants and authorised surrogates

Appendix 12: Informed consent materials

- Revised PIS(v6); PIS 2 (non-randomised VOT arm – v1)
- Revised PIS Credit Card Size (v5)
- Revised Consent Form (v6); Consent Form 2 (non-randomised VOT arm – v1)
- Revised Supplementary Consent Form (v2); Supplementary Consent Form 2 ((non-randomised VOT arm – v1)
- Revised Initial Script for Clinic Nurses (v5); Initial Script for Clinic Nurses 2 (non-randomised VOT arm – v1)
- Revised Letter to GPs (v3)
- VOT Participant Recording Procedure Booklet (v2)
- Revised Research Protocol (v5)

33) Biological specimens. Clinics will be asked to submit a sputum sample for smear and culture testing at the sites local microbiology laboratory (recommended good practice). The results of this will be reported to the study but the sample or subsequent culture will not be made available to the study. No other biological specimens will be collected.

VOT interim analysis plan

At an NIHR review of the programme grant in Dec 2015, the slower than expected recruitment rates in the VOT study were reviewed along with preliminary data on the acceptability of VOT vs DOT, uptake of either intervention following randomization and cross-over rates from DOT to VOT arms and vice versa.

These early indicators are suggestive that the primary outcome (the proportion achieving over 80% of doses that were planned to be observed being observed) is likely to be favourable for VOT. As a result of this meeting, NIHR asked for an interim analysis to be conducted that would examine whether there is evidence for interventional effectiveness at this stage potentially allowing the study to be stopped early or to inform recommendations about how long the trial needed to continue.

When writing the trial protocol for the study an interim analysis was not considered necessary due to the low risk of adverse outcomes in this trial. This document therefore sets out a plan for an interim analysis as requested by NIHR, the funders of the study. The results of this interim analysis will be reviewed by the trials independent monitoring committee who will advise NIHR about early termination of the trial. This document sets out an a-priori plan for the interim analysis.

The following primary and secondary outcomes will be examined:

Primary Outcome Measure:

Proportion of participants having more than 80% of scheduled VOT/DOT sessions successfully completed in the 2 months following randomisation (binary aggregation).

Secondary Outcome measure:

Proportion of doses observed over 2 months (continuous variable)

Primary and secondary analyses

It is proposed that the interim analysis be conducted in two parts. The first is based upon the full study protocol written before the study began, and the second is proposed as it will provide evidence of effectiveness more relevant to implementation of the intervention outside of the RCT setting.

- Primary analysis – as described above. Intention to treat (ITT) analysis of primary outcome. (i.e. considering all those who were randomized regardless of whether they ever took up either arm of the intervention).
- Secondary analysis including only those individuals who started the randomized intervention and have at least 1 week of outcome data for the primary outcome and secondary outcome (this is because a number of patients refused observation immediately following randomization).

Sensitivity analyses

VOT readings were classified in the following way:

1. All meds observed
2. Some meds observed
3. No meds observed
4. Unknown, unable to tell
5. Other
6. Probably took meds
7. Technical issues with clip

The Probably category includes instances when we have evidence that the patient sent a video clip but it could not be opened (patients have no control over whether or not a sent video clip can be opened)

DOT observations were classified in the following way:

1. All meds observed
2. Some meds observed
3. No meds observed

4. Unknown, unable to tell
5. Other

6. Probably took meds
7. Self-observed therapy

The self observed therapy indicates times when the case worker believes the patient took their medicine but did not observe this.

For each of the analytic strategies described above there will be one main analysis and two sensitivity analyses. The main analysis will consider VOT 1 or 6 and DOT 1 or 6 as positive outcomes (i.e comparing how often sessions were thought to have been definitely or probably successfully completed).

Sensitivity analysis A) VOT 1 and DOT 1 will be considered as positive outcomes i.e. comparing how often sessions were definitely completed successfully.

Sensitivity analysis B) VOT 1, 2, 6 or 7 and DOT 1, 2, 6 or 7 will be considered as positive outcomes i.e. considering self-observed sessions in DOT patients to be considered as successful treatment.

Primary outcome analysis strategy:

For the primary outcome, the following combination of analyses will be conducted:

- Analysis 1: Primary ITT analysis (positive outcomes: VOT 1 or 6; DOT 1 or 6)
- Analysis 2: Primary ITT analysis (sensitivity analysis A - positive outcomes: VOT 1; DOT 1)
- Analysis 3: Primary ITT analysis (sensitivity analysis B - positive outcomes: VOT 1, 2, 6 or 7; DOT 1, 2, 6 or 7)
- Analysis 4: Secondary analysis restricted to only those individuals who started the randomized intervention and have at least 1 week of outcome (positive outcomes: VOT 1 or 6; DOT 1 or 6)
- Analysis 5: Secondary analysis restricted to only those individuals who started the randomized intervention and have at least 1 week of outcome (sensitivity

analysis A - positive outcomes: VOT 1; DOT 1)

- Analysis 6: Secondary analysis restricted to only those individuals who started the randomized intervention and have at least 1 week of outcome (sensitivity analysis B - positive outcomes: VOT 1, 2, 6 or 7; DOT 1, 2, 6 or 7)

Descriptive analysis of the baseline characteristics of those randomised to intervention and control arms will be compared to check for balanced randomisation. Logistic regression will be used for all analyses of the primary outcome for analyses 1-3. All analyses will account for the balanced randomisation by inclusion of time since start of treatment variable (binary, less than two months or not). We will also adjust for treatment clinic, age and sex a-priori, as we believe these to be prognostic factors. If there is evidence of imbalance in randomisation a multivariable logistic regression analysis will be reported separately to account for this. As we are primarily examining the effect of social rather than biological factors within this study, we will not include disease, or microbiological factors. Instead any adjustment will consider social risk factors including, problem drug or alcohol use, no recourse to public funds, homelessness, imprisonment, mental health, and history of non-adherence or previous treatment, and immigration status. As analyses 4-6 are not being conducted on an ITT basis, we expect there to be imbalance within the study arms and are likely to require additional adjustment to control for these confounding factors.

Secondary outcome analysis strategy:

For the secondary outcome, the following combination of analyses will be conducted:

- Analysis 1: Primary ITT analysis (positive outcomes: VOT 1 or 6; DOT 1 or 6)
- Analysis 2: Primary ITT analysis (sensitivity analysis A - positive outcomes: VOT 1; DOT 1)
- Analysis 3: Primary ITT analysis (sensitivity analysis B - positive outcomes: VOT 1&2; DOT 1 or 6 or 7)
- Analysis 4: Secondary analysis restricted to only those individuals who started the randomized intervention and have at least 1 week of outcome (positive outcomes: VOT 1 or 6; DOT 1 or 6)

- Analysis 5: Secondary analysis restricted to only those individuals who started the randomized intervention and have at least 1 week of outcome (sensitivity analysis A - positive outcomes: VOT 1; DOT 1)

Analysis 6: Secondary analysis restricted to only those individuals who started the

randomized intervention and have at least 1 week of outcome (sensitivity analysis B - positive outcomes: VOT 1, 2, 6 or 7; DOT 1, 2, 6 or 7)

Descriptive analysis of the baseline characteristics of those randomised to intervention and control arms will be compared to check for balanced randomisation. Logistic regression will be used for all analyses of the primary outcome for analyses 1-3. All analyses will account for the balanced randomisation by inclusion of time since start of treatment variable (binary, less than two months or not). We will also adjust for treatment clinic, age and sex a-priori, as we believe these to be prognostic factors. If there is evidence of imbalance in randomisation a multivariable linear regression analysis will be reported separately to account for this. As we are primarily examining the effect of social rather than biological factors within this study, we will not include disease, or microbiological factors. Instead any adjustment will consider social risk factors including, problem drug or alcohol use, no recourse to public funds, homelessness, imprisonment, mental health, and history of non-adherence or previous treatment, and immigration status. As analyses 4-6 are not being conducted on an ITT basis, we expect there to be imbalance within the study arms and are likely to require additional adjustment to control for these confounding factors.

Stopping rules

We are planning one interim analysis and will therefore use the Haybittle–Peto boundary as a rule for deciding whether to stop the trial early. We will use a p-value threshold = 0.001 for this purpose.

The recommendation on whether to stop the trial early will be informed by this p-value threshold for analyses 1-6 of the primary and secondary outcomes. As we believe that many individuals did not take up the offer of DOT post-randomisation, for the

purposes of early stopping we will place more emphasis on analyses 3-6 for the primary outcome when making a decision as to whether to terminate the trial early or not.

Analysis of possible harms

The interim analysis will include a descriptive analysis of the possible harms, including:

- Loss to follow up levels
- Deaths from tuberculosis
- Reported side effects

Additional notes on the classification of outcomes

When participants are in hospital their dose will be considered to have been observed. When participants are in prison or custody the dose will be considered to have been observed if it can be verified with offender health that they were aware of the treatment regime. When patients are out of the country VOT participants will be encouraged to continue to take VOT clips which can either be submitted via a wifi connection or will automatically submit on return to the UK. Doses due during time abroad will not be considered as part of the primary outcome for either arm, but will be described to highlight the potential value.

ADDITIONAL REFERENCES

1. White P, Jit M, Stagg H, Pimpin L, Choi Y, Mugwagwa T. Economic analysis of identifying and managing tuberculosis in hard to reach groups: homeless and prison populations. NICE, 2011.
2. Curtis L, Burns A. Unit Costs of Health and Social Care: Personal Social Services Research Unit; 2016. Available from: http://www.pssru.ac.uk/project-pages/unit-costs/2016/
